# *Arabidopsis* choline transporter-like 1 (CTL1) regulates secretory trafficking of auxin transporters to control seedling growth

**DOI:** 10.1371/journal.pbio.2004310

**Published:** 2017-12-28

**Authors:** Yuan Wang, Lei Yang, Yumei Tang, Renjie Tang, Yanping Jing, Chi Zhang, Bin Zhang, Xiaojuan Li, Yaning Cui, Chunhua Zhang, Jisen Shi, Fugeng Zhao, Wenzhi Lan, Sheng Luan

**Affiliations:** 1 State Key Laboratory for Pharmaceutical Biotechnology, Nanjing University-Nanjing Forestry University Joint Institute for Plant Molecular Biology, College of Life Sciences, Nanjing University, Nanjing, China; 2 Department of Plant and Microbial Biology, University of California, Berkeley, Berkeley, California, United States of America; 3 College of Life Sciences, Northwest University, Xi’an, Shanxi, China; 4 Key Laboratory for Genetics and Breeding of Forest Trees and Ornamental Plants, Ministry of Education, College of Biological Sciences and Technology, Beijing Forestry University, Beijing, China; 5 Department of Botany and Plant Pathology, Purdue University, West Lafayette, Indiana, United States of America; 6 Nanjing University–Nanjing Forestry University Joint Institute for Plant Molecular Biology, Key Laboratory of Forest Genetics and Biotechnology, Nanjing Forestry University, Nanjing, China; University of California San Diego, United States of America

## Abstract

Auxin controls a myriad of plant developmental processes and plant response to environmental conditions. Precise trafficking of auxin transporters is essential for auxin homeostasis in plants. Here, we report characterization of *Arabidopsis CTL1*, which controls seedling growth and apical hook development by regulating intracellular trafficking of PIN-type auxin transporters. The *CTL1* gene encodes a choline transporter-like protein with an expression pattern highly correlated with auxin distribution and is enriched in shoot and root apical meristems, lateral root primordia, the vascular system, and the concave side of the apical hook. The choline transporter-like 1 (CTL1) protein is localized to the trans-Golgi network (TGN), prevacuolar compartment (PVC), and plasma membrane (PM). Disruption of *CTL1* gene expression alters the trafficking of 2 auxin efflux transporters—*Arabidopsis* PM-located auxin efflux transporter PIN-formed 1 (PIN1) and *Arabidopsis* PM-located auxin efflux transporter PIN-formed 3 (PIN3)—to the PM, thereby affecting auxin distribution and plant growth and development. We further found that phospholipids, sphingolipids, and other membrane lipids were significantly altered in the *ctl1* mutant, linking CTL1 function to lipid homeostasis. We propose that CTL1 regulates protein sorting from the TGN to the PM through its function in lipid homeostasis.

## Author summary

Auxin, a plant hormone, controls many aspects of plant growth and development. The precise transport and distribution of auxin hold the key to its function. A number of transport proteins are known to be involved in auxin translocation, and the PIN proteins, which are an integral part of membranes in plants, play a pivotal role in this process. Several PIN proteins are localized in the plasma membrane to mediate auxin efflux from cells, but their regulation is not well known. In this report, we analyze the role of a choline transport protein, choline transporter-like 1 (CTL1), and find that it controls the trafficking of *Arabidopsis* PM-located auxin efflux transporter PIN-formed 1 (PIN1) and *Arabidopsis* PM-located auxin efflux transporter PIN-formed 3 (PIN3) to the plasma membrane, thereby regulating auxin distribution during plant growth and development. In addition, we show that CTL1 has a role in lipid homeostasis in the membrane; thus, these findings provide a mechanistic link between choline transport, lipid homeostasis, and vesicle trafficking in plants. We conclude that CTL1 is a new factor in secretory protein sorting and that this finding contributes to the understanding of not only auxin distribution in plants but also protein trafficking in general.

## Introduction

Dynamic endomembrane trafficking delivers proteins and other cargo molecules to a variety of organelles and thus controls almost all aspects of plant development and physiology, including gravitropism, epidermis differentiation, guard cell movement, cell wall remodeling, defense against pathogens, and hormone signaling [1]. In this context, exocytosis and endocytosis determine the abundance and dynamics of signaling receptors and transporters at the plasma membrane (PM), which in turn afford cells the ability to respond to extracellular stimuli. For example, PM-located auxin transporters PIN-formed (PINs) and AUXIN RESISTANT 1 (AUX1) have been shown to continuously cycle between PM and endosomal compartments, resulting in dynamic changes in localization and abundance at the PM, where they function to control auxin gradients between cells. Many components have been shown to affect the trafficking and PM abundance of auxin transporters. These include the nucleotide exchange factor for ADP-ribosylation-factor-type GTPases (ARF-GEFs) [2–4], retromer proteins [5], adaptor protein complex 3 (AP-3) [6, 7], endosomal sorting complexes [8, 9], the trans-Golgi network/early endosome (TGN/EE) protein ECHIDNA [10], and several protein kinases [11–13]. The trafficking regulators safeguard the polarity and dynamics of auxin transporters, making possible the spatiotemporal variations in auxin distribution that act as an instructive signal for a wide variety of cellular events, ranging from polarity establishment during the earliest phases of plant embryogenesis to other morphogenetic events, including root development and the formation of the apical hook during seedling development [2–5, 10–14]. Because of the dynamics of these auxin transporters in the endomembrane system, they can be used as cargo proteins to study the regulation of the membrane trafficking process in cell biology. In genetic analysis, the mutations in genes encoding these transporters are often trackable because the mutants often show severe phenotypic changes in plant growth and development.

In a screen for mutants defective in phloem development, Dettmer et al. [15] identified choline transporter-like 1 (CTL1) to be essential for sieve tube formation and thus overall plant development. The *CTL1* gene encodes a protein with multiple transmembrane domains that has been shown to mediate choline transport [15]. However, the cellular mechanism for CTL1 function in plant development remains unknown. In tracking down the mechanism of CTL1 function, we discovered that CTL1 is closely associated with auxin signaling in the control of plant developmental processes. We further found that disruption of CTL1 function impaired endomembrane trafficking of several auxin transporters and probably some other unidentified cargo proteins as well. CTL1 acts at both the secretory vesicles (SVs) and the clathrin-coated vesicles (CCVs) of the TGN to regulate the trafficking of PIN1 and PIN3, which may explain the phenotypes of the *ctl1* mutant in seedling growth and apical hook development. As a choline transporter, CTL1 plays a crucial role in the homeostasis of membrane lipids, including phospholipids and sphingolipids. As lipid compositions of cell membranes influence vesicular transport, CTL1 function provides the mechanistic link between choline transport, lipid remodeling, and vesicular trafficking of PM proteins. We conclude that CTL1 is a previously unrecognized regulator of endomembrane trafficking in plants.

## Results

### A null mutant lacking CTL1 displays a cell elongation defect

A significant portion of plant genomes encode proteins with multiple transmembrane domains that may function as transporters. However, a large majority of these open reading frames (ORFs) are designated as “proteins of unknown function.” We took a comprehensive reverse genetic approach to identify the function of these proteins by screening *Arabidopsis* SALK transfer deoxyribonucleic acid (T-DNA) insertion lines disrupting the expression of corresponding genes. One of the lines, *SALK_065853C* in Columbia-0 (Col-0) background, was recovered for its severely stunted growth phenotype. This line contains a T-DNA insertion in the *At3g15380* gene, previously named *CHOLINE-TRANSPORTER-LIKE 1* (*CTL1)* for its biochemical function as a choline transporter and its function in sieve tube formation [15].

**Fig 1:**
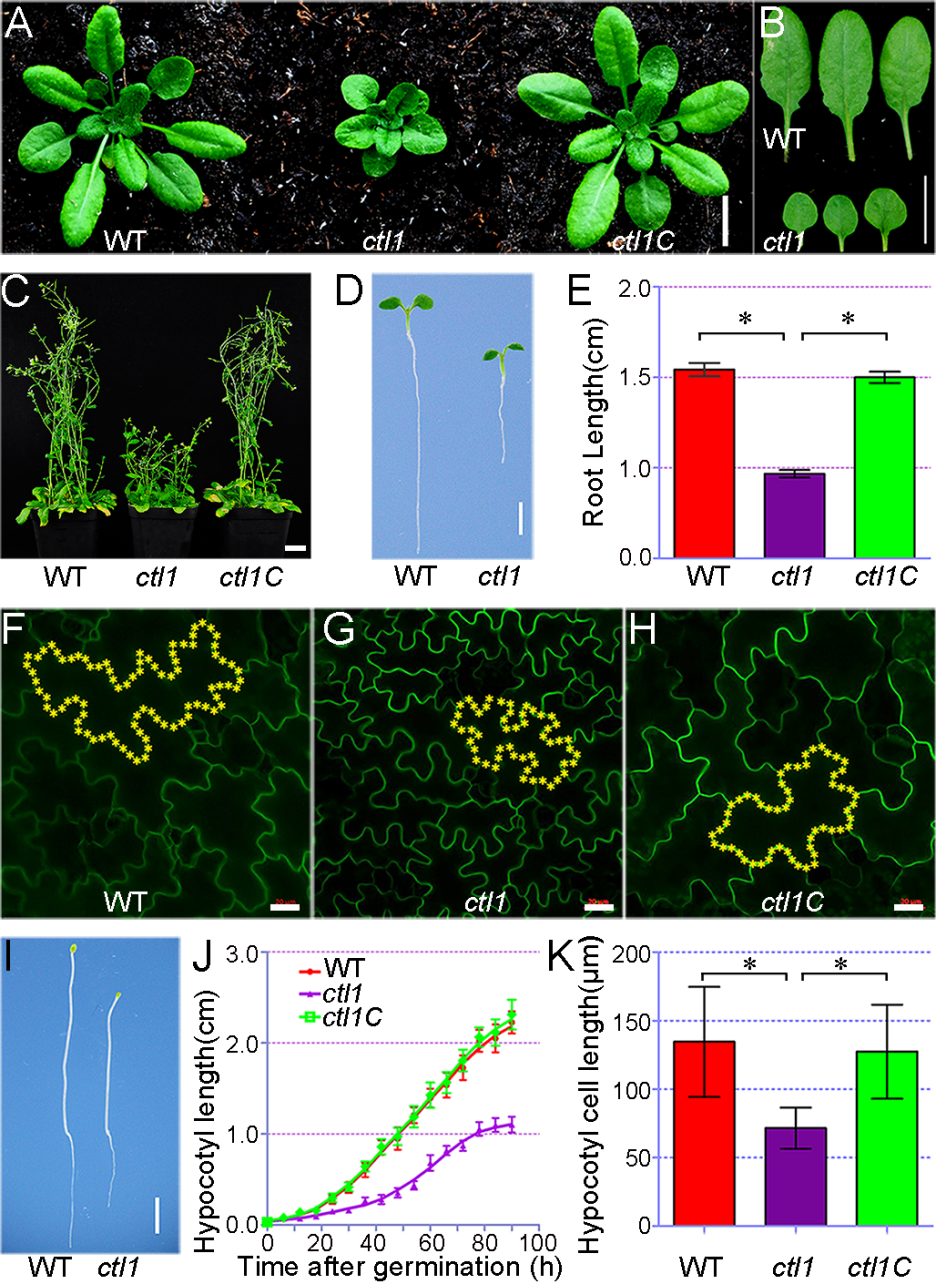
The *CTL1*-knockout mutant has a defect in cell elongation. (A) The growth phenotype of wild-type (WT), *ctl1* mutant, and complementation lines transformed with At3g15380 genomic DNA (*ctl1C*). After being grown on half-strength Murashige and Skoog (MS) medium for 7 days, the seedlings were transferred to the soil and grown for 23 days before photographs were taken. Bar = 1 cm. (B) The rosette leaves from 30-day-old *ctl1* mutant as compared to those of the WT. Bar = 1 cm. (C) The dwarfed phenotype of 2-month-old adult *ctl1* plants compared to the WT and *ctl1C*. Bar = 2 cm. (D) Five-day-old WT and *ctl1* mutant grown on half-strength MS medium. Bar = 3 mm. (E) The root length of 5-day-old WT, *ctl1*, and *ctl1C*. Three independent experiments were performed. Data are mean ± SD. *n* = 6 for each genotype. Significant differences are indicated as *P < 0.05 (Student *t* test). (F–H) Comparison of epidermal cells in the cotyledon of 5-day-old WT (F), *ctl1* (G), and *ctl1C* seedlings (H). Representative cells were highlighted with yellow asterisks. Scale bars in panels F–H are 20 μm. (I) Growth phenotype of 5-day-old etiolated WT and *ctl1* seedlings. Bar = 2 mm. (J) Hypocotyl length of etiolated WT, *ctl1*, and *ctl1C* seedlings. Data are mean ± SD. *n* = 5 for each genotype. (K) The hypocotyl cell length of 5-day-old etiolated WT, *ctl1*, and *ctl1C* seedlings. Data are mean ± SD, and significant differences are indicated as *P < 0.05 (*n* = 30, Student *t* test). The raw data for panels E, J, and K can be found in S1 Data.

The insertion in *CTL1* occurred in the first intron (281 bp from the start codon) to generate a transcriptional knockout (S1A–S1C Fig). We backcrossed the *ctl1* mutant with the wild type for 2 generations to segregate other possible T-DNA insertions and found that the isolated homozygous *ctl1* mutant lacking other insertions consistently showed the typical *ctl1* phenotype observed with the original SALK line. We further confirmed that the phenotype of the *ctl1* mutant resulted from lack of CTL1 function by complementation using a genomic fragment of the *CTL1* gene and a CTL1- enhanced green fluorescent protein (EGFP) fusion construct driven by the CTL1 promoter, respectively (S1D Fig and S2A Fig). The transgenic plants expressing *CTL1* genomic fragment or CTL1-EGFP fusion protein in the *ctl1* mutant background displayed a similar growth phenotype to that of the wild-type plants (Fig 1A, S1E Fig and S2D Fig), supporting the conclusion that disruption of the *CTL1* gene rendered the dwarf phenotype of the mutant plants.

We examined the *ctl1* phenotype in detail and found that 30-day-old *ctl1* seedlings grown in the soil displayed smaller, round, and dark green epinastic leaves with short petioles (Fig 1A and 1B and S1F–S1H Fig) and that 2-month-old adult *ctl1* mutants exhibited a dwarfed phenotype (Fig 1C). To examine root growth, we planted seeds on half-strength Murashige and Skoog (MS) agar medium and found that 5-day-old *ctl1* seedlings had shorter roots as compared to the wild type (Fig 1D and 1E), consistent with previous observations [15].

We further examined the cell size in both wild-type and mutant plants and found that the stunted growth phenotype in the *ctl1* mutant resulted from a defect in cell elongation. To determine cell size, we expressed AUX1-GFP in wild-type, *ctl1*, and complemented lines (*ctl1C*) so that the cell periphery lit up with the GFP for confocal imaging. The images collected from confocal laser scanning microscopy revealed that the size of leaf epidermal cells in *ctl1* was smaller than that of those in the wild type and *ctl1C* (Fig 1F–1H). Typically, the cell size in the *ctl1* mutant plants was only 49% of the wild type (S1 Table). As hypocotyl cells elongate quickly without cell division, hypocotyls grown in the dark are excellent models for examining cell elongation [16]. We thus analyzed the hypocotyl length of etiolated seedlings and found that the hypocotyls of *ctl1* mutant seedlings elongated more slowly compared with the wild type and *ctl1C* (Fig 1I and 1J). Moreover, the epidermal cell length of *ctl1* hypocotyls was only about half of that of the wild type (Fig 1K). Taken together, these results demonstrate that CTL1 is an essential regulator of cell elongation in *Arabidopsis*. This finding explains an overall dwarf phenotype of the *ctl1* mutant seedlings beyond the defect in phloem development observed earlier [15].

### CTL1 is localized to the PM, TGN, and prevacuolar compartment (PVC)

**Fig 2:**
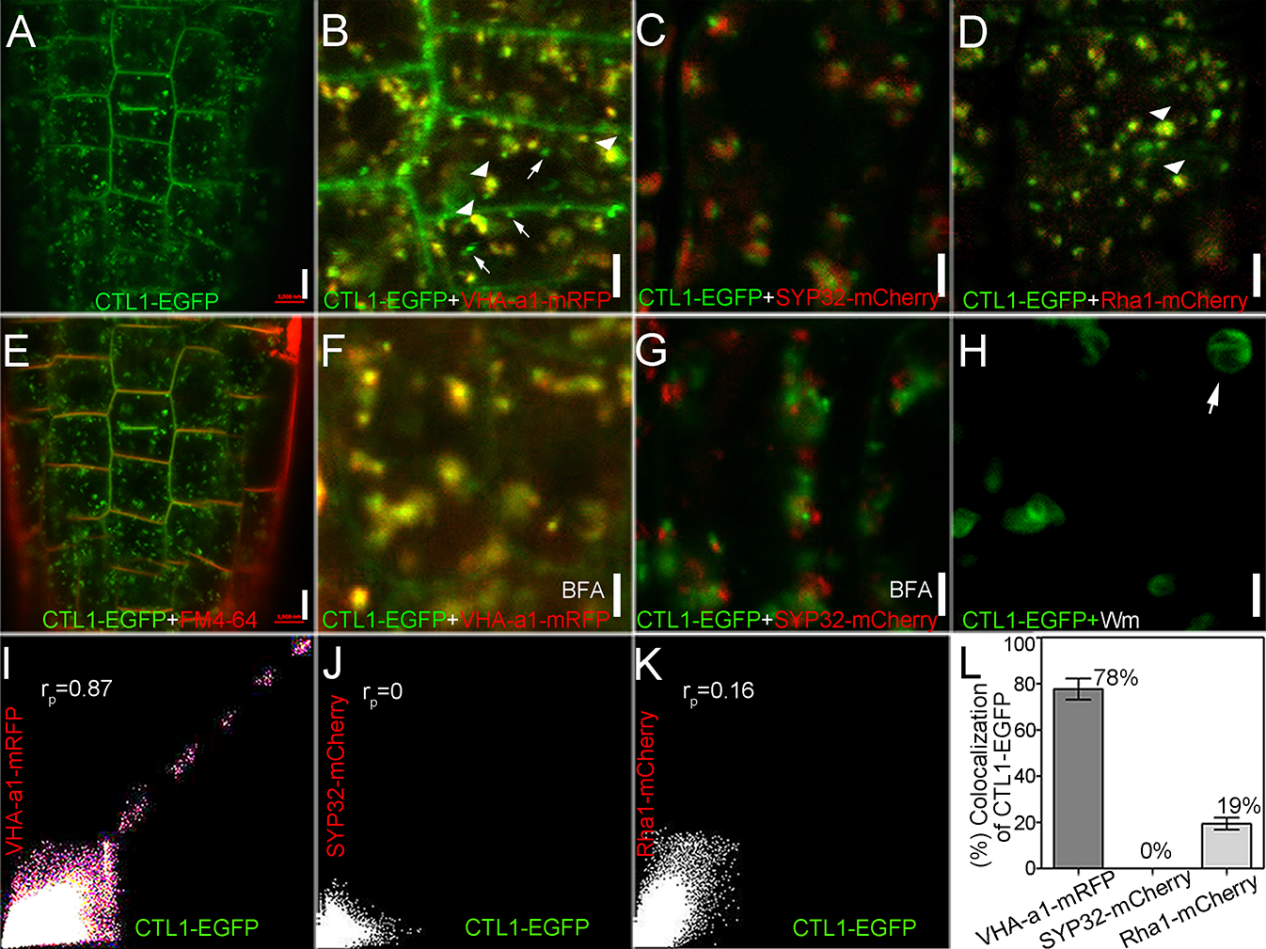
Subcellular localization of choline transporter-like 1 (CTL1). (A) CTL1- enhanced green fluorescent protein (EGFP) signals in root cortex cells of CTL1-EGFP transgenic plants expressing CTL1-EGFP in the *ctl1* mutant background. Bar = 5 μm. (B–D) Comparison of CTL1-EGFP (green) signal with subcellular markers tagged with monomer red fluorescent protein (mRFP) or mCherry (red). Bar = 2 μm. (B) CTL1-EGFP is largely colocalized with the trans-Golgi network/early endosome (TGN/EE) marker, vacuolar proton ATPase subunit VHA-a isoform 1 (VHA-a1)-mRFP. Arrowheads and arrows indicate overlapping and nonoverlapping signals, respectively. (C) CTL1-EGFP is localized to distinct compartments from Syntaxin of plants 32 (SYP32)-mCherry. (D) Partial colocalization of CTL1-EGFP with *Arabidopsis* Rab homolog F2A (Rha1)-mCherry. Arrowheads indicate overlapping signals. (E) Root cortex cells labeled with FM4-64 (red) and CTL1-EGFP (green). The image was captured within 5 minutes of incubation with 4 μM FM4-64. Bars = 5 μm. (F) Colocalization of CTL1-EGFP and VHA-a1-mRFP in root cortex cells treated with brefeldin A (BFA). (G) CTL1-EGFP and SYP32-mCherry are not colocalized in BFA-treated cells. The images in (F) and (G) were captured after the roots were treated with 100 μM BFA for 60 minutes. Bars = 2 μm. (H) CTL1-EGFP signals in root cells after they were treated with 33 μM wortmannin (Wm) for 60 minutes. White arrow shows the ring-like structure. Bars = 2 μm. (I–K) Colocalization analysis of CTL1-EGFP with VHA-a1-mRFP (I), SYP32-mCherry (J), or Rha1-mCherry (K) using the ImageJ software with the colocalization finder plugin. The linear Pearson correlation coefficient (r_p_) indicates the percentage of signal overlap, and an r_p_ value of 1.0 represents 100% colocalization. (L) The colocalization percentage of CTL1-EGFP with VHA-a1-mRFP, SYP32-mCherry, or Rha1-mCherry. Data are mean ± SD. Six images acquired from 3 roots were used. The raw data for panel L can be found in S1 Data.

To further study the cellular mechanism of CTL1 function, we determined the subcellular localization of CTL1 in root cortex cells of *Arabidopsis* seedlings by using a *CTL1-EGFP* transgenic line in the *ctl1* mutant background. Real-time quantitative reverse transcription PCR (qRT-PCR) assay showed that this transgenic line had levels of *CTL1* transcript that were similar to those in the wild-type plants (S2B Fig). Furthermore, the CTL1-EGFP construct in this transgenic line complemented the dwarfed phenotype of the *ctl1* mutant (S2D Fig), indicating that this line expressed functional CTL1-EGFP fusion proteins in the same compartments as the native CTL1 protein. Therefore, we used this line to track down the subcellular location of CTL1-EGFP protein using confocal microscopy. The fluorescence signal generated by CTL1-EGFP fusion appeared at the PM and also displayed a punctate pattern in the cytosol (Fig 2A and S2E Fig). To identify intracellular punctate structures at which CTL1-EGFP is localized, we crossed this *CTL1-EGFP* transgenic line with the transgenic lines that stably express fluorescent markers for several organelles. We found that most of the punctate signals of CTL1-EGFP colocalized with vacuolar proton ATPase subunit VHA-a isoform 1 monomer red fluorescent protein (VHA-a1-mRFP) (Fig 2B), a TGN/EE marker [17]. The linear Pearson correlation coefficient (r_p_) for the colocalization was 0.87 (Fig 2I). We did not observe colocalization of CTL1-EGFP fluorescence signals with Syntaxin of plants 32 (SYP32)-mCherry (r_p_ = 0) (Fig 2C and 2J), the Golgi apparatus marker [18]. In addition, some CTL1-EGFP fluorescence signals overlapped with those of *Arabidopsis* Rab homolog F2A (Rha1)-mCherry (r_p_ = 0.16) (Fig 2D and 2K), the PVC marker [18]. We conducted more experiments to further examine the subcellular localization of CTL1. First, we stained root tips with FM4-64 for a short time to label the PM [19] and found that the CTL1-EGFP fluorescence colocalized with the FM4-64 signal (Fig 2E). Secondly, we treated root tips with fungal toxin brefeldin A (BFA), which blocks trafficking from the TGN to the PM, leading to the formation of enlarged endosomal compartments referred to as BFA bodies [20]. We detected both CTL1-EGFP and VHA-a1-mRFP fluorescence in the BFA bodies (Fig 2F). However, the CTL1-EGFP and SYP32-mCherry signals did not overlap in the presence of BFA (Fig 2G). Finally, we treated root tips with wortmannin (Wm), a drug that preferentially triggers the swelling of PVC but has little effect on the morphology of the Golgi apparatus and the TGN/EE [21]. After 1 hour of treatment with 33 μM Wm, the morphology of some organelles labeled by CTL1-EGFP became swollen and showed a PVC-specific ring-like structure (Fig 2H), indicating that CTL1 is also sorted to the PVC.

We also quantified the colocalization of CTL1-EGFP with various organelle markers in root tip cells of 4-day-old seedlings. This analysis revealed a large proportion (78% ± 4.6%) of colocalization between CTL1-EGFP and the TGN marker VHA-a1-mRFP, a smaller proportion (19% ± 2.6%) of colocalization between CTL1-EGFP and the PVC marker Rha1-mCherry, and no colocalization with SYP32-mCherry (Fig 2L). The TGN is subdivided into 2 structure forms, SVs and CCVs [22]. We also detected that CTL1-EGFP colocalizes with mCherry-clathrin light chain 2 (CLC2) (S3 Fig). As VHA-a1 and CLC2 reside in the SVs and the CCVs, respectively [22], colocalization with both VHA-a1-mRFP and mCherry-CLC2 illustrated that CTL1 is localized to both subdomains of the TGN.

### Postembryonic expression of CTL1

**Fig 3:**
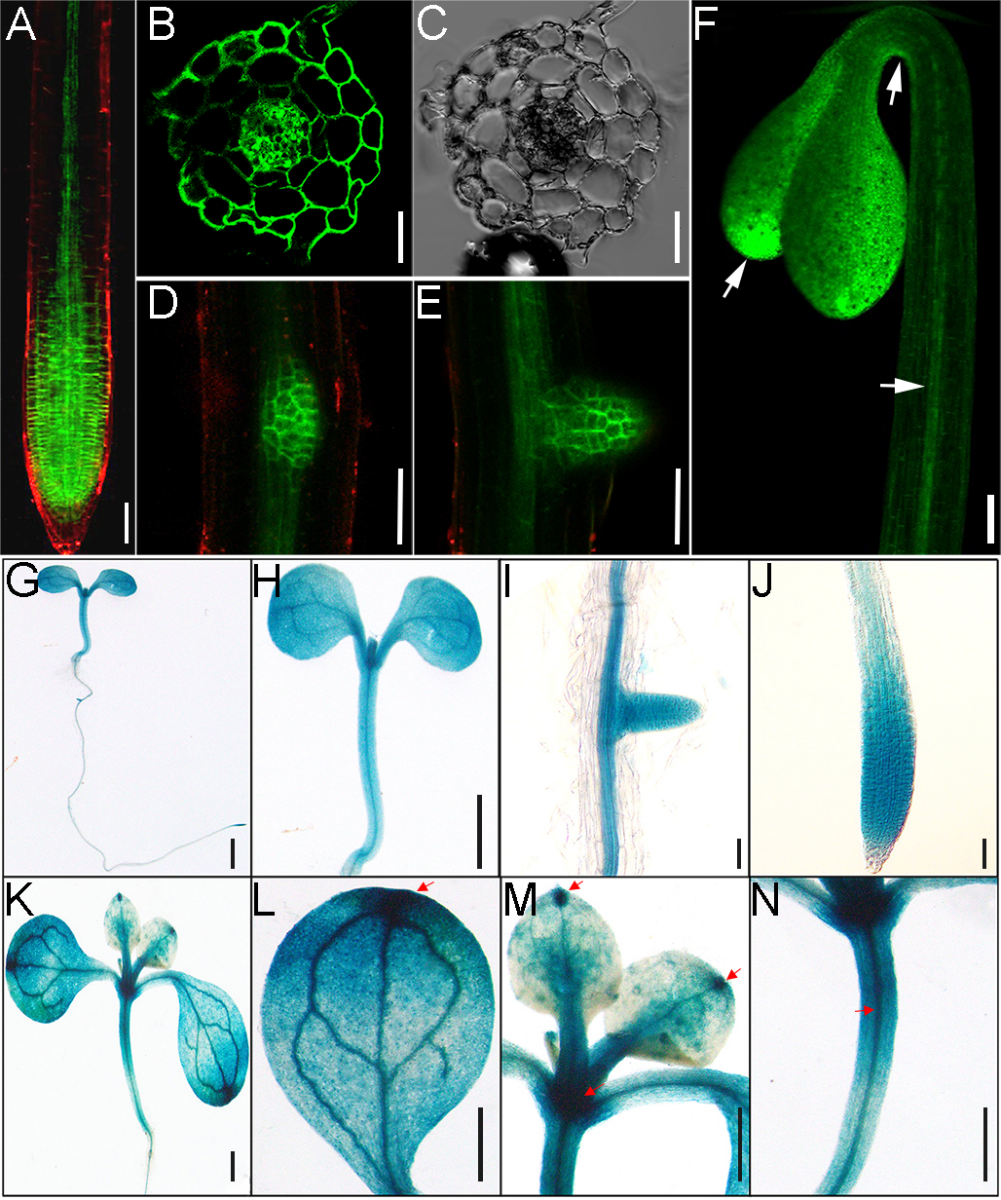
Expression pattern of *CTL1* gene reporters in *Arabidopsis* seedlings. (A–F) Green fluorescence signals in transgenic plants harboring the *CTL1-EGFP* construct. Choline transporter-like 1 enhanced green fluorescent protein (CTL1-EGFP) signals were detected in the primary root (A), lateral root primordium (D), and emerged lateral root (E) of 5-day-old seedlings (Bars = 50 μm). The transection view of CTL1-GFP at the GFP channel (B) and the differential interference contrast (DIC) channel (C) shows GFP signal in all cell types in the meristem region of a primary root from a 5-day-old seedling. Bars = 25 μm. (F) CTL1-EGFP signals in a 3-day-old etiolated seedling. Arrows indicate the sites of strong expression. Bars = 1 mm. (G–N) Histochemical analysis of *CTL1* promoter–β-glucuronidase (GUS) expression in transgenic plants. (G–J) GUS staining of a 5-day-old seedling (G), showing expression in shoot (H), emerging lateral root (I), and primary root (J). (K–N) GUS staining of a 7-day-old seedling (K), showing expression in cotyledon (L), the first pair of true leaves (M), and the hypocotyl (N). The scale bars in (I) and (J) are 100 μm, and other bars are 1 mm.

We used the *CTL1-EGFP* transgenic line to study the expression pattern of *CTL1*, since the expression of CTL1-EGFP was driven by the *CTL1* promoter region (S2 Fig). The GFP fluorescence was ubiquitously distributed in the meristem and elongation zones but restricted to vascular tissues in the mature zone of roots (Fig 3A). Cross sections through the root meristem zone displayed the distribution of CTL1-EGFP signal in all cell layers (Fig 3B and 3C). Furthermore, GFP was also highly expressed in the newly initiated lateral roots (Fig 3D and 3E).

In dark-grown seedlings, *CTL1* was expressed in the whole cotyledon with enrichment at the blade tips, the vasculature of the hypocotyl, and the concave side of the apical hook (Fig 3F). When grown on the half-strength MS medium under dark conditions, *Arabidopsis* apical hook development proceeds through 3 phases: the hook formation phase, the maintenance phase, and the opening phase [10, 23, 24]. CTL1-EGFP signals appeared at both the convex and the concave sides of the hook, with the maximum in the concave side (S4G and S4H Fig), and the expression at the concave side gradually disappeared during the opening phase (S4A–S4F and S4J Fig), suggesting that CTL1 function may be associated with apical hook development.

To further confirm the expression pattern of *CTL1*, we generated transgenic plants harboring the CTL1pro:CTL1:GUS construct in which the EGFP reporter was replaced by the β-glucuronidase (GUS) reporter. In the apical hook region, CTL1-GUS shows the same expression pattern as the CTL1-EGFP in the concave side of the apical hook (S4I Fig). In the roots of 5-day-old seedlings, GUS activity was detected in the similar tissues labeled by EGFP signals (Fig 3G*–*3J). In shoots of 7-day-old seedlings, the expression pattern of CTL1 was ubiquitous, but a relatively higher level of GUS activity was detected in the apical meristem, at the tips of cotyledon and true leaves, and in vascular tissues (Fig 3K*–*3N), largely overlapping with the GFP signals detected earlier (Fig 3F).

### CTL1 loss-of-function mutant shows defects associated with auxin

**Fig 4:**
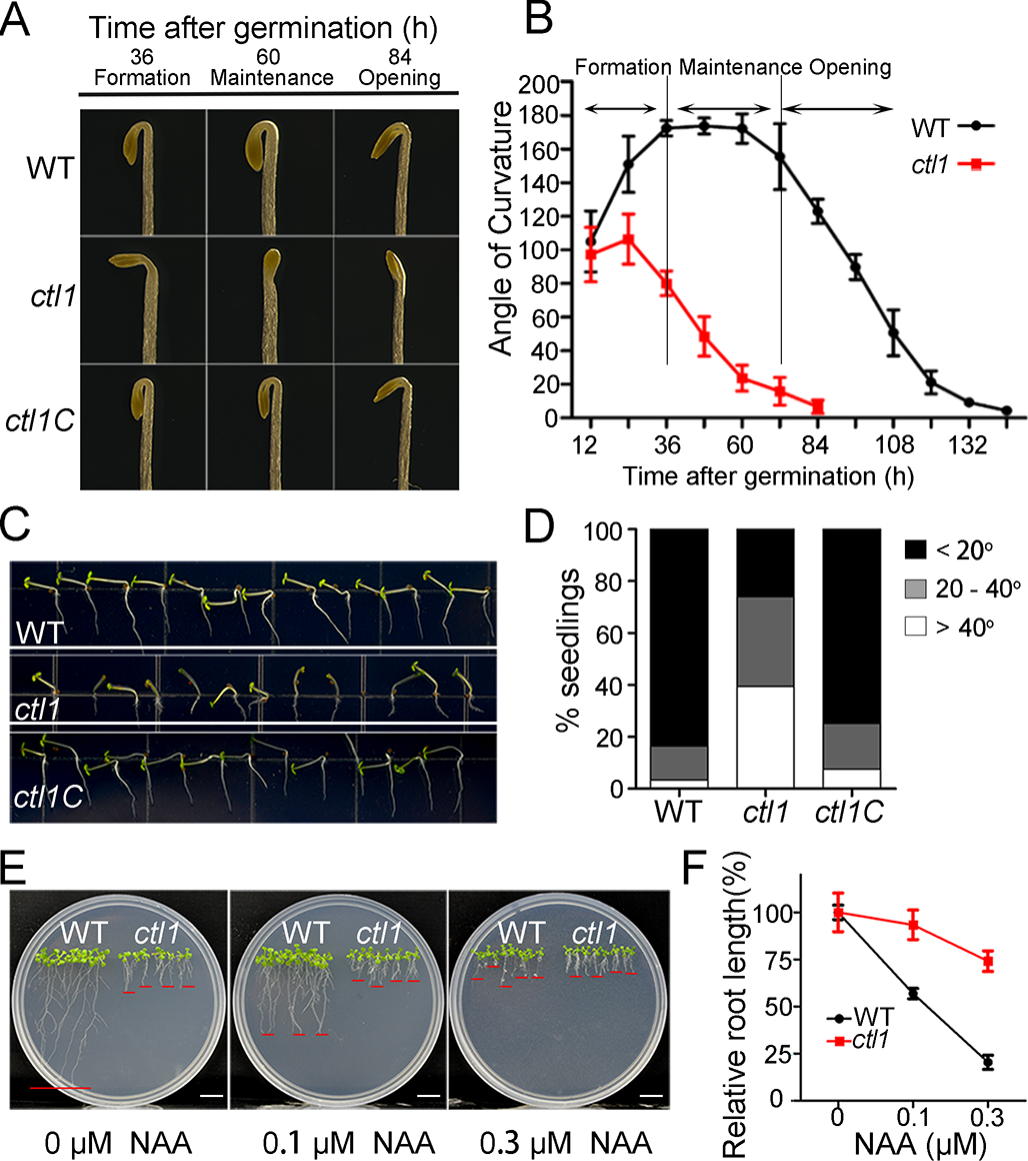
The *ctl1* mutant shows auxin-related phenotypic changes. (A) Representative images of the apical hooks of the wild type (WT), *ctl1*, and the *ctl1* complementation line (*ctl1C*) at 36, 60, and 84 hours after germination in the dark. (B) Kinetic analysis of apical hook angles of etiolated seedlings of WT, *ctl1*, and *ctl1C* during apical hook development. Data are mean ± SD of 4 replicate experiments. (C) Representative images of phototropic growth of WT, *ctl1*, and *ctl1C* seedlings. After being vertically grown on half-strength Murashige and Skoog (MS) medium in the dark for 2 days, the seedlings were exposed to unilateral light for 2 days before the photographs were taken. (D) Quantitation of the phenotypes shown in panel C. The bending angles of the hypocotyls towards the horizontal direction were measured using the ImageJ software. The percentages of seedlings in each of 3 categories (<20°, 20–40°, and >40°) were calculated. WT, *n* = 116; *ctl1*, *n* = 119; and *ctl1C*, *n* = 119. (E) Growth phenotype of 10-day-old WT and *ctl1* mutant plants grown on half-strength MS medium containing 0, 0.1, or 0.3 μM 1-naphthylacetic acid (NAA). Bars = 1 cm. (F) The *ctl1* mutant shows reduced NAA sensitivity. Relative root length (%) indicates shortening of the primary roots as a result of NAA in the medium. Data are mean ± SD. Three independent experiments were performed. *n* = 6 for each experiment. The raw data for panels B, D, and F can be found in S1 Data.

Phenotypic analyses of the *ctl1* mutant in detail provided insights into CTL1 function in growth and development. The *ctl1* mutant showed reduced primary root growth, whereas the lateral roots reached approximately the same length as the primary root, apparently resulting from lack of apical dominance [15]. In addition, we found that the *ctl1* showed a severe defect in apical hook development, consistent with high levels of expression of CTL1 in the apical hook (Fig 3F and S4 Fig). In particular, etiolated *ctl1* seedlings never formed the fully closed apical hook, and the hook quickly opened without an apparent maintenance phase (Fig 4A and 4B).

*ctl1* defects in cell elongation (described earlier) and defects in primary and lateral root growth all point to relevance with the auxin response. Furthermore, apical hook development is the result of differential cell elongation mediated by uneven auxin distribution [23]. These auxin-related phenotypes of *ctl1* prompted us to investigate the connections between CTL1 and auxin. We went on exploring whether there are other auxin-related phenotypes altered in the *ctl1* mutant. One process we studied was phototropism, a well-characterized light response mediated by asymmetric auxin distribution in plants [4]. As shown in Fig 4, *ctl1* had a reduced hypocotyl phototropic response to unilateral light as compared with the wild type and the complemented line (Fig 4C and 4D). In addition, we also found that *ctl1* seedlings were less sensitive to high levels of 1-naphthylacetic acid (NAA) that inhibit root elongation. Compared to the wild type, *ctl1* roots were resistant to auxin inhibition over a range of auxin concentrations (0, 0.1, and 0.3 μM) (Fig 4E and 4F). We also tested the sensitivity to salicylic acid (SA) and found that both the wild type (WT) and the *ctl1* mutant were sensitive to SA (S5 Fig), unlike the situation with auxin. All these findings support the hypothesis that CTL1 is involved in auxin-related processes.

### Auxin distribution is altered in the *ctl1* mutant

**Fig 5:**
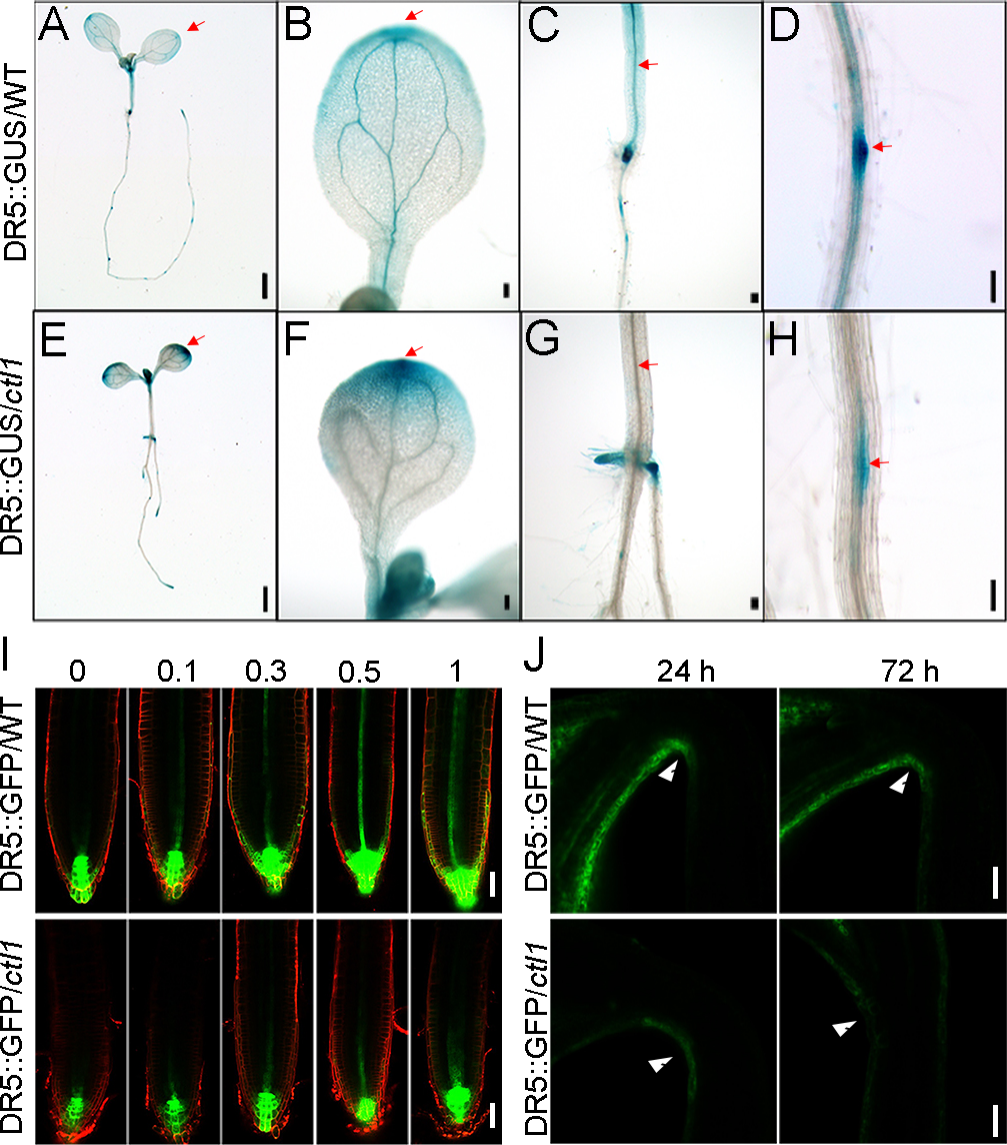
The *ctl1* mutant shows altered auxin distribution pattern. (A–H) DR5::GUS expression pattern in 7-day-old wild type (DR5::GUS/WT) (A–D) and *ctl1* mutant (DR5::GUS/*ctl1*) (E–H). The red arrows point to the areas where signals are different between DR5::GUS/WT and DR5::GUS/*ctl1*. Scale bars in panel A and panel E are 1 mm, and those in panels B–D and F–H are 100 μm. (I) Expression pattern of DR5::GFP in the wild type (DR5::GFP/WT) (upper panel) and the *ctl1* mutant (DR5::GFP/*ctl1*) (lower panel) treated with 0, 0.1, 0.3, 0.5, or 1 μM 1-naphthylacetic acid (NAA). Scale bars are 50 μm. (J) DR5::GFP signals in the concave side of the apical hook of the wild type (WT) and the *ctl1* mutant at 1 and 3 days after germination. The red arrows show the sites where green fluorescent protein (GFP) signals were accumulated in the WT but not in the mutant. Scale bars are 20 μm. DR5, DIRECT REPEAT5; GUS, β-glucuronidase.

To study auxin distribution in the *ctl1* mutant, we crossed it with a transgenic line harboring DR5::GUS, a reporter of auxin levels [5]. In the *ctl1* mutant background, the DIRECT REPEAT5 (DR5)-driven GUS expression was greatly enhanced in the cotyledons, especially at the tips, but was dramatically reduced in the vascular tissues of the hypocotyls and roots, as compared to the pattern of DR5::GUS in the WT background (Fig 5A–5H and S6A Fig). In the roots, the DR5::GFP signal in the WT and the *ctl1* mutant showed a similar distribution pattern, with a condensed signal in the primary root tips. However, after being treated with NAA (0.1, 0.3, 0.5, and 1 μM) for 12 hours, the DR5::GFP signal was enhanced in the meristem and elongation zones of WT roots with increasing NAA concentrations, but the level of this response was reduced in *ctl1*, suggesting reduced transport or reduced response of auxin (Fig 5I and S6B Fig). Moreover, a maximum DR5::GFP signal was detected in the concave side of the apical hooks of the WT, but this was largely diminished in the mutant (Fig 5J and S6C Fig).

### CTL1 controls the abundance of PIN1 and PIN3 in the root and the apical hook

**Fig 6:**
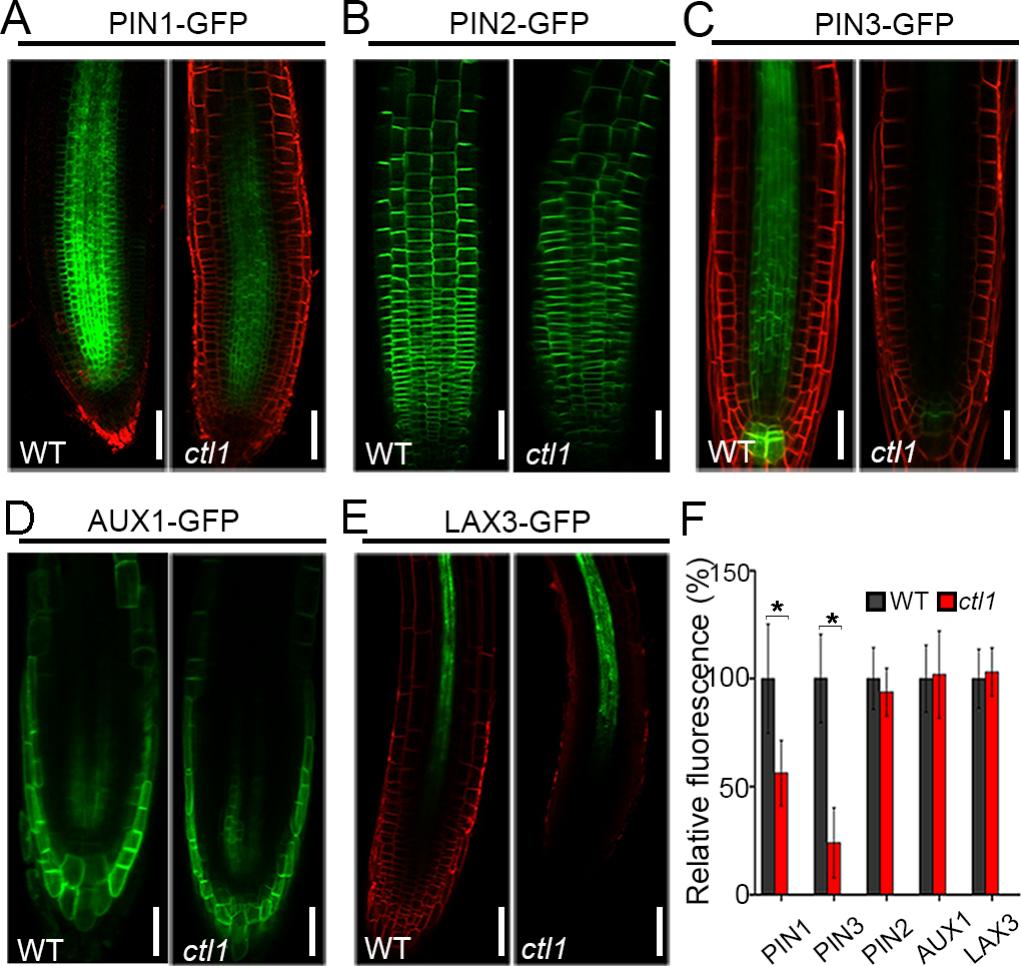
The *ctl1* mutant shows reduced levels of *Arabidopsis* PM-located auxin efflux transporter PIN-formed 1 (PIN1) and *Arabidopsis* PM-located auxin efflux transporter PIN-formed 3 (PIN3). The green fluorescent protein (GFP) signals indicate the abundance of several auxin transporters, including PIN1pro:PIN1:GFP (A), PIN2pro:PIN2:GFP (B), PIN3pro:PIN3:GFP (C), AUX1pro:AUX1:GFP (D), and LAX3pro:LAX3:GFP (E), in 4-day-old primary root of the wild type (WT) and the *ctl1* mutant. The red signals are from FM4-64 staining. Scale bars are 50 μm. (F) Relative abundance of auxin transporters (PIN1, PIN2, PIN3, AUXIN RESISTANT 1 [AUX1], and Like AUX1 protein 3 [LAX3]) in *ctl1* mutant is shown as % of the WT. Data are mean ± SD from 91, 64, 44, 39, and 40 cells in 5 roots of the transgenic lines expressing PIN1-GFP, PIN3-GFP, PIN2-GFP, AUX1-GFP, and LAX3-GFP, respectively. Three independent experiments were performed. Asterisks indicate a significant difference (*P < 0.05, Student *t* test). The raw data for panel F can be found in S1 Data.

We showed that disruption of CTL1 function appears to affect auxin transport and distribution (Figs 4 and 5). Together with the finding of CTL1 subcellular localization to the PM and endomembranes (Fig 2), we speculated that CTL1 may regulate the trafficking of PM-localized proteins so that distribution of auxin transporters may be altered in the *ctl1* mutant. In order to test this hypothesis, we examined the expression and localization of several auxin transporters in the *ctl1* mutant by using auxin transporter-GFP markers. We crossed the *ctl1* mutant with transgenic lines containing PIN1pro:PIN1:GFP, PIN2pro:PIN2:GFP, PIN3pro:PIN3:GFP, AUX1pro:AUX1:GFP, or LAX3pro:LAX3:GFP and monitored the GFP signals by confocal microscopy. We found a dramatically reduced accumulation of PIN1-GFP and PIN3-GFP in the *ctl1* mutant as compared to the WT. More specifically, the abundance of PIN1-GFP in the mutant was about half (56% ± 14.2%) of the WT, and for PIN3 it was only a quarter (26% ± 19.3%) of the WT (Fig 6A, 6C and 6F). However, other auxin transporters, including PIN2, AUX1, and Like AUX1 protein 3 (LAX3), were not affected by the *CTL1* mutation (Fig 6B and 6D–6F). We also found that the brassinosteroid receptor BRI1 showed no difference in the WT and the *ctl1* mutant (S8 Fig). Because PIN-mediated auxin efflux also plays a major role during apical hook development [24, 25], we also examined GFP signal in the apical hook and again found reduced levels of PIN1-GFP and PIN3-GFP, but not of AUX1-GFP, in the *ctl1* mutant as compared to the WT (S7 Fig). The loss of PIN3 can cause an enhanced DR5 signal in the cotyledons of *Arabidopsis* [25]. As we also detected an enhanced DR5 signal in the cotyledons of *ctl1* mutant, we proposed that reduced abundance of PIN1 and PIN3 may lead to changed auxin transport and distribution (shown by DR5::GUS/GFP lines earlier) that in turn causes changes in auxin-related phenotypes in the *ctl1* mutant.

To determine whether the reduced abundance of PIN1 and PIN3 proteins in the *ctl1* mutant results from a decrease in the transcript level, we examined the RNA levels of auxin transporter genes in the WT and the *ctl1* mutant. The qRT-PCR analysis showed that the mRNA levels of *PIN1* and *PIN3* in *ctl1* were slightly higher than those in the WT (S9 Fig), suggesting that down-regulation of PIN1 and PIN3 proteins in *ctl1* occurs at the post-transcriptional level.

### CTL1 regulates trafficking of PIN1 and PIN3 to the PM

**Fig 7:**
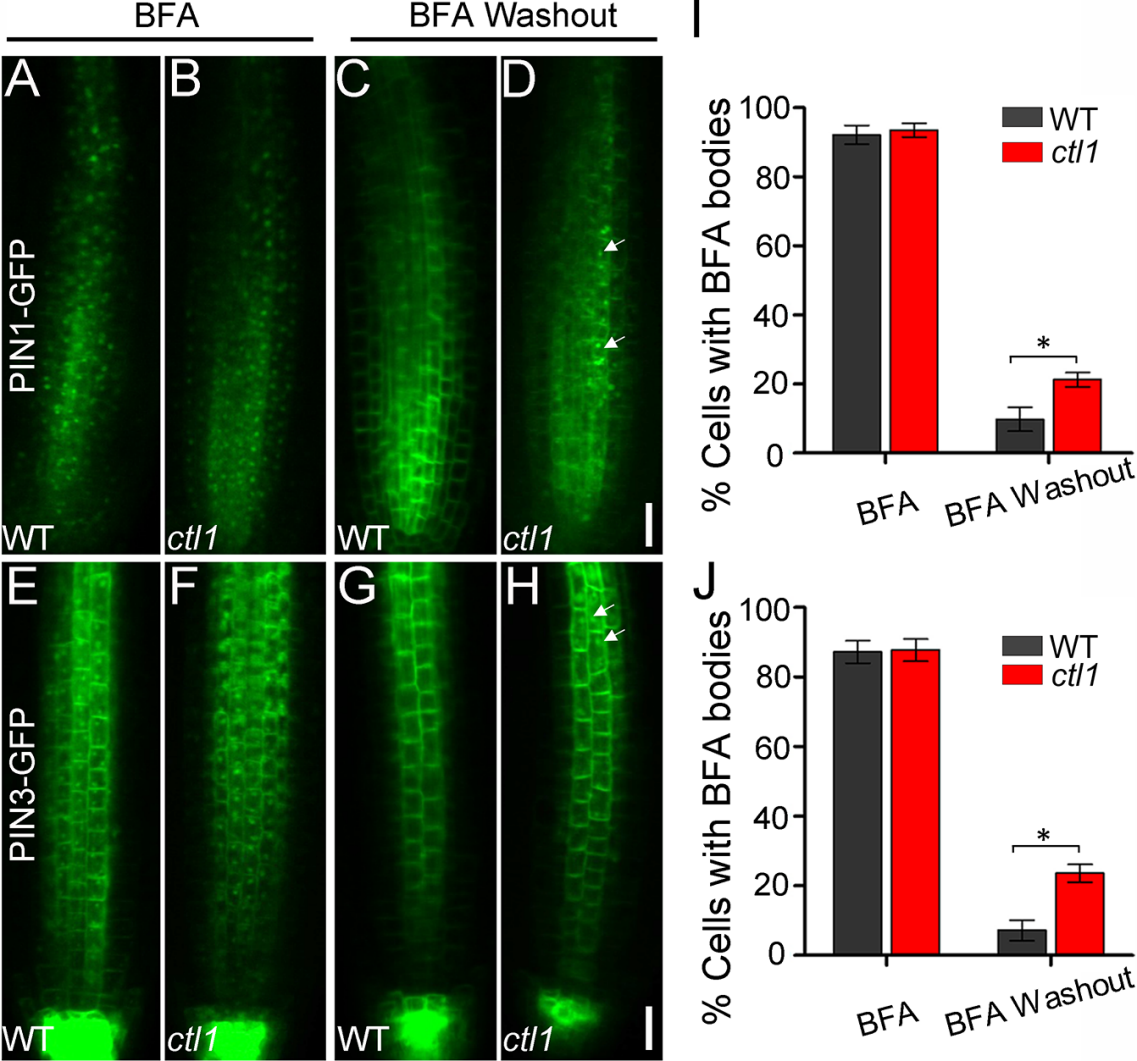
Choline transporter-like 1 (CTL1) affects *Arabidopsis* PM-located auxin efflux transporter PIN-formed 1 (PIN1) and *Arabidopsis* PM-located auxin efflux transporter PIN-formed 3 (PIN3) recycling. (A–D) PIN1-green fluorescent protein (GFP) localization in wild-type (WT) (A and C) and *ctl1* roots (B and D) treated with brefeldin A (BFA) for 60 minutes before (A and B) or after (C and D) washout for 90 minutes. Insets at the lower right corner of panels A–D show enlarged views of PIN1-GFP labeled BFA bodies (from areas indicated by arrows). Scale bars are 20 μm. (I) Percentage of cells with PIN1-GFP labeled BFA bodies before and after BFA washout in the WT and the *ctl1* mutant. Data are mean ± SD. Three independent experiments were performed. Five roots were measured for each experiment (Student *t* test, *P < 0.05). (E–H) PIN3-GFP localization in WT (E and G) and *ctl1* (F and H) roots treated with BFA as shown for PIN1-GFP. Scale bars are 20 μm. (J) Percentage of cells with PIN3-GFP labeled BFA bodies before and after BFA washout in the WT and the *ctl1* mutant. Data are mean ± SD. Three independent experiments were performed. Five roots were used for each experiment (Student *t* test, *P < 0.05). The raw data for panels I and J can be found in S1 Data.

The reduced abundance of PIN1 and PIN3 in the *ctl1* mutant, together with CTL1 localization to the TGN, suggests that CTL1 protein may affect the recruitment of PINs from the TGN to the PM. In the study of protein trafficking, BFA is often used to block protein sorting from the TGN to the PM and to induce protein internalization from the PM to the TGN. Thus, BFA treatment of cells results in the formation of large endosomes called BFA bodies that contain PM-destined proteins. These BFA bodies are rapidly disintegrated, and the proteins are targeted back to the PM again after BFA washout [26–28]. Therefore, BFA washout has been widely used to measure the speed of trafficking from the TGN back to the PM [26–29]. We analyzed the trafficking of PIN1-GFP and PIN3-GFP using a similar approach. Upon treatment of WT and *ctl1* roots with 50 μM BFA for 60 minutes, similar percentages of cells with BFA bodies were detected in the WT and the *ctl1* mutant (Fig 7A, 7B and 7I). However, after BFA washout for 90 minutes, we detected 9.7% and 21.6% of root cells containing PIN1-GFP-decorated BFA bodies in the WT and *ctl1*, respectively (Fig 7C, 7D and 7I). Similarly, about 7.6% and 23.5% of cells contained PIN3-GFP-labeled BFA bodies in the WT and *ctl1*, respectively (Fig 7E–7H and 7J). We also did the BFA washout experiment using the PIN2-GFP/WT and PIN2-GFP/*ctl1* transgenic lines and found no difference between the WT and the *ctl1* mutant (S10 Fig). These data indicate that loss of CTL1 causes a delay of trafficking in PIN1 and PIN3 proteins, which may in turn lead to the auxin-related developmental defects we observed in the *ctl1* mutant plants.

### CTL1 plays a vital role in the homeostasis of membrane lipids in *Arabidopsis*

**Fig 8:**
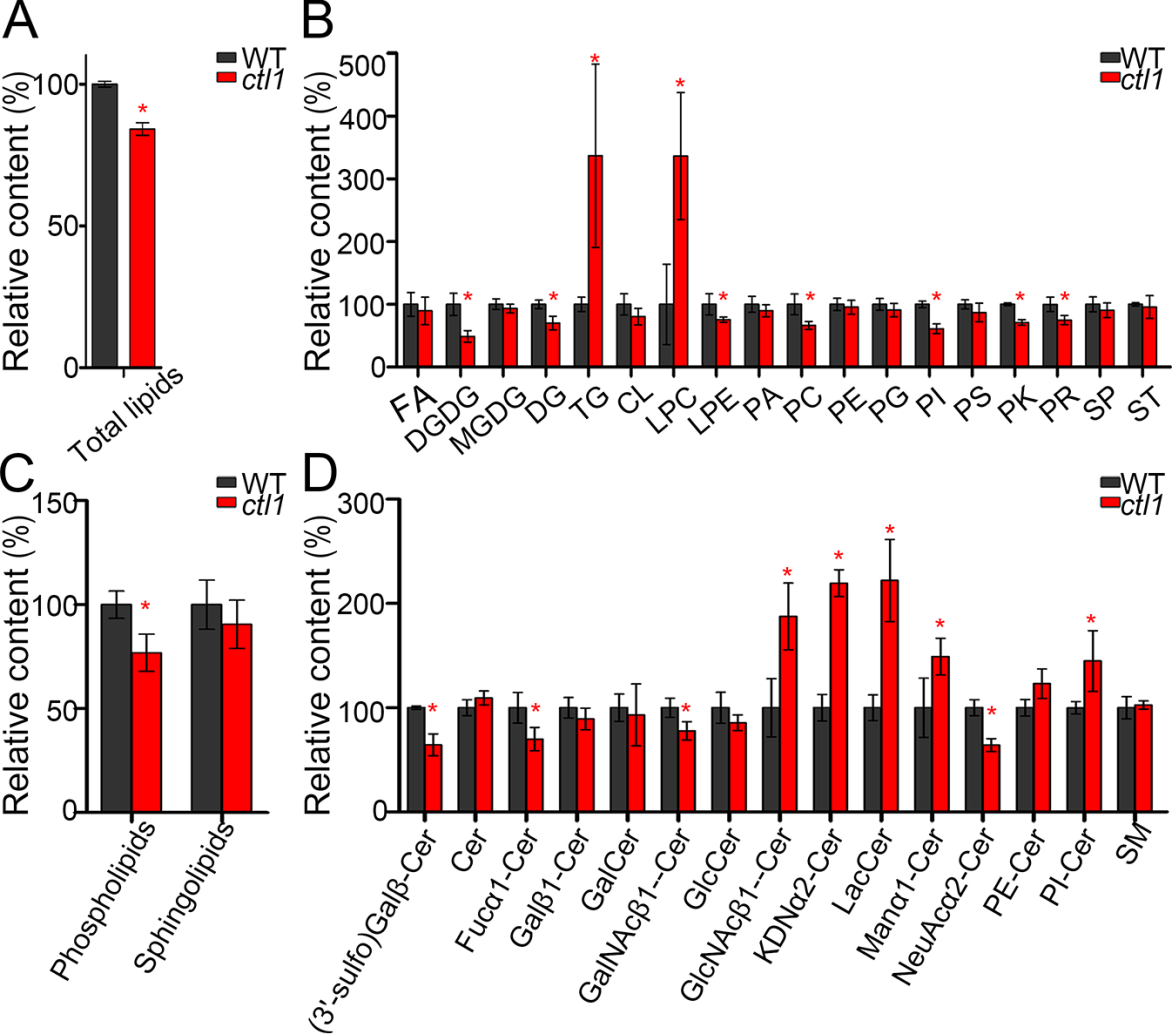
The lipidomics analysis of wild type and *ctl1*. (A) The relative content of total lipids in the wild type (WT) and the *ctl1* mutant. (B) The changes in the subclasses of lipids in the *ctl1* mutant. Fatty acyls (FAs), digalactosyldiacylglycerol (DGMG), monogalactosyldiacylglycerol (MGDG), diacylglycerol (DG), triacylglycerol (TG), lysophosphatidylcholine (LPC), lysophosphatidylethanolamine (LPE), phosphatidic acid (PA), phosphatidylcholine (PC), phosphatidylethanolamine (PE), phosphatidylglycerol (PG), phosphatidylinositol (PI), phosphatidylserine (PS), polyketides (PKs), prenol lipids (PRs), sphingolipids (SPs), and sterol lipids (STs). (C) The relative intensity of total glycerophospholipids and SPs in the WT and the *ctl1* mutant. (D) Some subclasses of SPs are significantly altered in the *ctl1* mutant. These include a group of ceramides (Cer) such as Fucα1-Cer: Fucα1-2GalNAcβ1-4(NeuAcα2-8NeuAcα2–3) Galβ1-4Glcβ-Cer; Galβ1-Cer: Galβ1-3GalNAcβ1-3Galα1-3Galβ1-4Glcβ-Cer; GalNAcβ1-Cer: GalNAcβ1-3Galα1-3Galα1-3Galα1-3Galα1-4Galβ1-4Glcβ-Cer; GlcNAcβ1-Cer: GlcNAcβ1-4Manβ1-4Glcβ-Cer; KDNα2-Cer: KDNα2-6Galβ1-4GlcNAcβ1-3Galβ1-4Glcβ-Cer; Manα1-Cer: Manα1-3Manβ1-4Glcβ-Cer; and NeuAcα2-Cer: NeuAcα2-8NeuAcα2-3Galβ1-4Glcβ-Cer. The classification of these SPs was from the method provided by the website LIPID Metabolites and Pathways Strategy(LIPID MAPS) (http://www.lipidmaps.org/). Data are mean ± SD. Four independent experiments were performed. Red asterisks indicate a significant difference (*P < 0.05, Student *t* test). The raw data for panels A–D can be found in S1 Data.

It has been reported that sphingolipids are involved in the trafficking of auxin transporters [30–32]. Choline is predominantly utilized for the synthesis of essential lipid components of the cell membranes, including sphingolipid [33]. A defect in choline transport may impair the homeostasis of these lipids, thereby affecting vesicle trafficking. To test if CTL1 functions in lipid homeostasis, we first monitored the lipid content in situ in the WT and mutant seedlings using Nile Red staining [32, 34]. The results showed that both polar and nonpolar lipids were reduced in the apical hook region of the *ctl1* mutant as compared to the WT (S11 Fig). To provide a more detailed analysis of lipid content, we used ultra-performance liquid chromatography coupled with mass spectrometry (UPLC-MS) to survey the lipid profiles of WT and *ctl1* plants. In the *ctl1* mutant, the total lipids were reduced to about 84% of the WT level (Fig 8A). In further analysis, we found that the glycerophospholipids (lysophosphatidylethanolamine, phosphatidylcholine, and phosphatidylinositol) in the *ctl1* mutant were reduced (Fig 8B). The content of total glycerophospholipids in *ctl1* mutant was about 77% of the level in the WT (Fig 8C). Although the total sphingolipid contents were similar between the WT and the *ctl1* mutant (Fig 8C), we found that the content of some specific types of sphingolipids were significantly altered in the *ctl1* mutant (Fig 8D). In particular, the abundance of sphingolipids containing very-long-acyl-chain fatty acids (C > 18 carbons) was altered in the *ctl1* mutant (S12 Fig), consistent with the earlier finding [31] that these sphingolipids are required for the targeting of specific auxin carriers to the PM.

To link the sphingolipids metabolism to CTL1 function further, we used the sphingolipid synthesis inhibitor fumonisin B1 (FB1) to treat the WT seedlings and found that this treatment induced apical hook defects as observed in the *ctl1* mutant (S13 Fig). Taken together, CTL1, as a choline transport protein, plays a role in homeostasis of lipids, including sphingolipids. The disturbed homeostasis of sphingolipids and other lipids in *ctl1* mutant may affect the delivery of PIN1, PIN3, and other cargos from the TGN to the PM.

## Discussion

### CTL1 is essential for auxin-regulated plant development

Auxin is the most investigated phytohormone and plays a pivotal role in virtually every aspect of plant growth and development, and such function often depends on its differential distribution within plant tissues [35]. At the whole plant level, auxin controls organogenesis, apical dominance, lateral root emergence, apical hook development and tropism, vascular development, and many other developmental processes [23, 36–39]. At the cellular level, auxin regulates cell elongation, cell division, and cell differentiation [40].

We showed that *Arabidopsis* CTL1 plays a key role in a number of auxin-regulated processes, including cell elongation, apical hook development, root morphology, and hypocotyl phototropic response to unilateral light in *Arabidopsis*. CTL1 expression corresponds to the auxin maxima in the shoot apical meristems, the tips of leaves, the lateral root primordia, the vascular system, and the concave side of apical hook. The dynamic changes in the CTL1 expression pattern strongly resemble the changes in auxin distribution during apical hook development [24]. Moreover, CTL1 is required for maintaining a normal pattern of DR5 expression, a marker for auxin distribution. Loss of function of CTL1 results in enhanced DR5 signals in the tips of cotyledons and reduced signals in the vascular system of hypocotyls, roots, and the concave side of the apical hook. The auxin-related phenotypes and altered auxin distribution observed in the *ctl1* mutant provide insights on the cellular and physiological processes that underpin the role of CTL1 in plant development.

### CTL1 affects the trafficking of auxin transporters to the PM

Auxin action depends on its differential distribution in plant tissues through a process called polar auxin transport [41]. A number of PM auxin transporters, such as PINs, AUX1, LIKE AUX1 (LAX), and subfamily B of the ATP-binding cassette proteins (ABCB), contribute to the influx and efflux of auxin and subsequently determine the distribution of auxin among different tissues and cells. Therefore, auxin homeostasis requires accurate subcellular targeting of these auxin transporters through secretory trafficking processes. In plant cells, the TGN is a central traffic hub responsible for protein trafficking in both biosynthetic and endocytic pathways. The endocytosed cargos from the PM were received by the TGN, and also through the TGN, biosynthetic cargos were sorted to the PM, cell wall, cell plate, PVC, tonoplast, and vacuole [42]. Protein composition and localization at the PM are regulated by exocytosis and endocytosis in response to developmental and environmental cues.

Here we provide strong evidence that CTL1 is located at multiple subcellular sites associated with secretory trafficking, including the TGN, PVC, and PM. As an endosomal protein, CTL1 may contribute to the trafficking of proteins from the TGN to the PM, especially those PM-localized auxin transporters, as supported by the finding that the abundance of some PIN-type auxin transporters was reduced in the *ctl1* mutant, which displays a number of auxin-related phenotypes. In particular, CTL1 is required for the efficient trafficking of PIN1 and PIN3 from the TGN to the PM. As a consequence of losing CTL1 function, the abundance of PIN1 and PIN3 is reduced, and auxin distribution is altered in the root and the apical hook. Further, the CTL1 expression pattern largely overlaps with those of PIN1 and PIN3 in roots and apical hooks [43] [44]. Loss of function of PIN1 or PIN3 causes defects in apical hook development, primary and lateral root growth, and response to unilateral light, which are also the defects found in the *ctl1* mutant [4, 45, 46]. Together, results on expression pattern, CTL1 contribution to PIN1 and PIN3 trafficking, and similar mutant phenotypes all support the conclusion that CTL1 regulates the trafficking of PIN1 and PIN3, thereby controlling auxin-related plant developmental processes.

### The mechanistic insights of CTL1 function in plant cells

CTL1 has been recently identified as a choline transporter required for sieve plate development [15], although the cellular mechanism underlying CTL1 function is not clear. Our discovery that CTL1 functions in promoting the trafficking of PIN1 and PIN3 may provide a mechanism for CTL1 function in auxin-related developmental processes, including previously described sieve plate biogenesis. In eukaryotic cells, lipid-dependent protein sorting is a major delivery mechanism for cargo proteins from the TGN to cell surface [47]. However, the mechanism by which lipids control vesicular trafficking remains unclear. As membrane lipids provide the microenvironment for protein actions, each membrane system consists of different domains with a specific composition of lipids, such as the sphingolipid-rich microdomains and lipid rafts. Recent studies also reveal various TGN subdomains with different sphingolipid and sterol compositions [48]. Choline is one of the precursors for the synthesis of membrane lipids, including phospholipids and sphingolipids. It is conceivable that CTL1, as a choline transport protein, would play a role in choline delivery to various cell membranes and thus modulate lipid composition of multiple organelles in plant cells. The defect in the homeostasis of membrane lipids in the *ctl1* mutant would cause the trafficking defects of some cargo proteins such as PIN1 and PIN3.

Although we cannot detect any differences between the WT and the *ctl1* mutant on the morphology, size, and number of the compartments labelled by different fluorescence-tagged organelle markers using confocal microscopy (S14 Fig), there might be other defects on the endomembrane system in *ctl1* that we were not able to identify. It remains unclear whether CTL1 functions as a transporter-like protein that prefers choline over other substrates [15]. Further investigations will be required to determine whether CTL1 transports choline specifically or can also transport other substrates. CTL1 is localized to different cellular compartments, including the TGN, PM, and PVC, and its trafficking is sensitive to BFA, suggesting that CTL1 itself is a cargo protein that goes through the secretory pathway. We found that CTL1 affects the trafficking of some special cargo proteins such as PIN1 and PIN3, but not PIN2. This indicates that CTL1 is not required for the proper function of global trafficking machinery; instead, it affects subgroups of proteins. Further survey on the cargo proteins that are affected by CTL1 and identification of proteins that interact with CTL1 will help further understanding of the mechanisms of CTL1 function.

## Materials and methods

### Plant materials and growth conditions

*Arabidopsis thaliana* ecotype Col-0 was used in this study. *Arabidopsis* WT, the T-DNA insertion line (SALK_065853C), and the transgenic lines expressing SYP32-mCherry (CS781677) or Rha1-mCherry (CS781672) were obtained from the *Arabidopsis* Biological Resource Center. The transgenic lines expressing VHA-a1-mRFP [17], DR5::GFP [49], DR5::GUS [50], PIN1pro:PIN1:GFP [51], PIN2pro:PIN2:GFP [52], PIN3pro:PIN3:GFP [52], mCherry-CLC2 [53], AUX1pro:AUX1:GFP [24], and BRI1pro:BRI1:GFP [29] have been described previously. Homozygous individuals were screened by PCR using the primers described in S2 Table. The surface-sterilized *Arabidopsis* seeds were plated on half-strength MS medium containing 1% (w/v) sucrose. The pH of the medium was adjusted to 5.7 and solidified using 1% (w/v) agar. For soil culture, 7-day-old *Arabidopsis* seedlings grown on half-strength MS medium were transferred to nutrient-rich soil (Pindstrup Mosebrug, Denmark). The seedlings were grown in a greenhouse under 150 μmol/m^2^/s light intensity with a 16-hour light/8-hour dark photoperiod at 22°C, unless otherwise indicated. For dark culture, the seeds were planted on half-strength MS and then transferred to white light for 6 hours to enhance germination; subsequently, the plates were wrapped with 2 layers of aluminum foil. For the unilateral light response experiment, seedlings were grown in darkness for 2 days and then exposed to unilateral white light (150 μmol/m^2^/s) for another 2 days, according to the published procedure [54].

### Pharmacological treatments

The seedlings were incubated in liquid half-strength MS medium containing a final concentration of 50 μM BFA (Sigma-Aldrich) or 33 μM wortmannin (Sigma-Aldrich) for 60 minutes, unless described otherwise in the figure legends. Labeling with FM4-64 (Molecular Probes) was carried out as described [29]. For BFA washouts, the seedlings were treated with 50 μM BFA for 1 hour, rinsed 3 times with water, and then incubated in half-strength MS liquid medium for 90 minutes before the imaging procedure.

### Root length, hypocotyl length, and apical hook curvature measurements

Images of individual seedlings were acquired using the OLYMPUS stereoscopic microscope equipped with a DP27 camera. The root length, hypocotyl length, and hook angle were then measured using the ImageJ software. The bending angles of the apical hook were scored as previously described [24]. For each time-lapse experiment, 6 seedlings were analyzed.

### Generation of plasmid constructs and transgenic *Arabidopsis* lines

For genetic complementation, a 6,107-bp genomic DNA fragment containing a 1,918-bp promoter region, a 3,117-bp coding region, and a 1,072-bp terminator region after the stop codon of the *CTL1* gene were amplified from WT genomic DNA and then cloned into the binary vector pCAMBIA-1300. To generate the *CTL1pro*:*CTL1*:*EGFP* construct, we fused the EGFP and NOS terminator sequence with the 5,032-bp genomic DNA of *CTL1*, including the 1,918-bp promotor region and the 3,114-bp coding region, and then cloned this recombinant DNA into the binary vector pCAMBIA-1300. A 10-alanine (Ala) linker sequence (GCT GCT GCC GCT GCC GCT GCG GCA GCG GCC) was also inserted between *CTL1* and *EGFP*. To generate the *CTL1pro*:*CTL1*:*GUS* construct, we fused the GUS and NOS terminator sequence to the CTL1 gene as described for the *CTL1pro*:*CTL1*:*EGFP* construct. The organization of these 2 constructs is shown in S1D and S2A Figs, respectively. To generate the *LAX3pro*:*LAX3*:*EGFP* construct, a 3,007-bp genomic DNA of *LAX3* including the promotor region and the coding region was fused with the EGFP and NOS terminator sequence and then cloned into pCAMBIA1300. The constructs were introduced into the *Agrobacterium tumefaciens* GV3101 strain for transformation into *Arabidopsis* by the floral dipping method [55]. The primers used for plasmids constructions are listed in S2 Table.

Transgenic lines expressing various markers including DR5::GFP, DR5::GUS, PIN1pro:PIN1:GFP, PIN2pro:PIN2:GFP, PIN3pro:PIN3:GFP, AUX1pro:AUX1:GFP, LAX3pro:LAX3:GFP, BRI1pro:BRI1:GFP, VHA-a1-mRFP, SYP32-mCherry, and Rha1-mCherry were individually crossed into the *ctl1* mutant background. Homozygous *ctl1* plants harboring various markers were isolated from F2 populations by scoring the specific phenotype of the mutant. The F3 and later generations were used for analyses. For colocalization analysis, transgenic lines expressing VHA-a1-mRFP, SYP32-mCherry, Rha1-mCherry, and mCherry-CLC2 were individually crossed with transgenic lines expressing CTL1pro:CTL1: EGFP.

### Confocal microscopy analysis

Imaging was performed on an LSM-710 confocal microscope (Zeiss) equipped with an argon/krypton laser. For the quantitative fluorescence intensity, confocal pictures were acquired using strictly identical acquisition parameters (laser power, photomultiplier, offset, zoom factor, and resolution) among the experimental seedlings. The excitation wave lengths for the GFP, FM4-64, mRFP, and mCherry signals were 488, 514, 543, and 587 nm, respectively. For colocalization analysis, the Colocalization Finder plugin of ImageJ was used. The linear Pearson correlation coefficient (r_p_) was used to indicate the extent of colocalization, with the value of +1.0 as complete colocalization. Detection of nonpolar lipids (excitation at 514 nm, emission at 520–560 nm) and polar lipids (excitation at 534 nm, emission at 600–700 nm) after Nile red staining was performed according to the published protocol [56].

### Histochemical GUS analysis

The staining was conducted according to the published protocol [57], with slight modifications. Briefly, transformed *Arabidopsis* seedlings were treated with 90% (vol/vol) acetone on ice for 30 minutes and then incubated in GUS staining solution (0.5 mM 5-bromo-4-chloro-3-indolyl-*β*-D-glucuronide, 100 mM Na_3_PO_4_, 10 mM EDTA, 0.1 mM K_3_[Fe(CN)_6_], 5 mM K_4_[Fe(CN)_6_] and 0.1% [vol/vol] Triton X-100, pH 7.0) at 37°C in darkness for 12 hours. After being sufficiently decolorized with 75% (vol/vol) ethanol, the plant tissues were photographed with an Olympus SZX12 microscope equipped with a camera.

### Quantitative real-time PCR analysis

Total RNA was extracted from plant samples using TRIzol reagent (Invitrogen), followed by synthesis of Poly (dT) complementary DNA using the M-MLV Reverse Transcriptase (Promega). qRT-PCR was performed using the SYBR Green I Master kit (Roche Diagnostics) according to the manufacturer’s instructions on a CFX Connect Real-Time System (Bio-Rad). All individual reactions were done in triplicate. The primers used for qRT-PCR are listed in S2 Table.

### Lipidomics analysis by UPLC-MS

Plant lipids from 5-day-old WT and *ctl1* mutant seedlings were extracted according to the published method [58], and the extracts were directly subjected to LC-MS analysis. The lipidomic data were recorded on an ESI-QTOF/MS (Xevo G2-S Q-TOF, Waters) coupled with a UPLC (ACQUITY UPLC *I*-Class system, Waters). Parameters for mass spectrometry were as follows: scan range, m/z 50–1,500; ion source, ESI; loop time, 0.2 seconds; cone voltages, 25 KV; capillary voltage, 3 KV; collision energy, 15–60 V; source temperature, 120°C; desolvation temperature, 400°C; desolvation gas flow, 500 L/h; and cone gas flow, 25 L/h. Raw data were imported into the commercial software Progenesis QI (Version 2.3) for data processing, including peak picking and acquiring compounds’ associated information such as m/z, retention time, and intensity. Next, data filtering was performed to delete low-quality data. R project was also applied in further data processing and statistical analysis.

## Supporting information

S1 Data Raw data for all figures and supplemental figures. (XLSX)

S2 Data The lipidomics raw data of the wild type and *ctl1*. (XLSX)

S1 Fig Isolation and complementation of the *CTL1* transfer deoxyribonucleic acid (T-DNA) insertional *A*. *thaliana* mutant. (A) Schematic representation of the *CTL1* gene. The T-DNA insertion site is located in the first intron (281-bp downstream of the *ATG* start codon) and is indicated by a triangle. Solid boxes and lines indicate the exons and introns, respectively. (B) Confirmation of the T-DNA insertion in the *ctl1* mutant by PCR using the forward primer (F) and reverse primer (R) of *CTL1* paired to the T-DNA border primer LB1. (C) Reverse transcription PCR (RT-PCR) analysis of *CTL1* mRNA from 10-day-old wild type (WT), *ctl1*, and *ctl1* complemented with a genomic fragment of the *CTL1* gene (*ctl1C*). *ACTIN2* mRNA was analyzed as a loading control. (D) Schematic model of the T-DNA region of vector pCAMBIA-1300 containing a genomic fragment of the *CTL1* gene. (E) The growth phenotype of 10-day-old WT, *ctl1*, and *ctl1C*. (F–H) Quantitative analysis of the petiole length of rosette leaves (F), the ratio of leaf length to width (G), and the rosette diameter (H) in 30-day-old WT, *ctl1* and *ctl1C* grown in the soil. Data are mean ± SD, and significant differences are indicated as ***P < 0.01 (*n* = 5, Student *t* test). The raw data for panels F–H can be found in S1 Data.
(TIF)

S2 Fig Complementation of the *ctl1* mutant with *CTL1pro*:*CTL1*:*EGFP* construct. (A) Schematic model of the transfer deoxyribonucleic acid (T-DNA) region of vector pCAMBIA-1300 containing a genomic fragment of the choline transporter-like 1 (CTL1) gene fused with enhanced green fluorescent protein (EGFP). A 10-alanine (Ala) linker sequence was inserted between CTL1 and EGFP. (B) Real-time quantitative reverse transcription PCR (qRT-PCR) analysis of CTL1 mRNA levels in 10-day-old wild type (WT), *ctl1*, and lines expressing CTL1pro:CTL1:EGFP in the *ctl1* mutant background. Relative expression levels were calculated as the ratio of CTL1 in various seedlings to that in WT. Data are mean ± SD (*n* = 3). Fifteen CTL1pro:CTL1:EGFP/*ctl1* lines were selected for qRT-PCR analysis, and one of them, #11, with CTL1 levels similar to those of the WT, was used for further studies. (C) RT-PCR analysis of CTL1 mRNA in 10-day-old *ctl1* mutant and CTL1pro:CTL1:EGFP-transformed line (CTL1pro:CTL1:EGFP/*ctl1* #11). ACTIN2 mRNA was analyzed as a loading control. (D) Growth phenotype of 5-day-old WT, *ctl1*, and CTL1pro:CTL1:EGFP/*ctl1* #11 grown on half-strength Murashige and Skoog (MS) solid medium. Bar = 2 mm. (E) Fluorescence signals in the primary root of a 4-day-old CTL1pro:CTL1:EGFP/*ctl1* #11 seedling grown on half-strength MS solid medium. Bar = 50 μm. The raw data for panel B can be found in S1 Data.
(TIF)

S3 Fig The subcellular location of choline transporter-like 1 enhanced green fluorescent protein (CTL1-EGFP) and mCherry-clathrin light chain 2 (CLC2) in a transgenic line expressing both *CTL1-EGFP* and *mCherry-CLC2*. A representative confocal image of CTL1-EGFP signals (A), mCherry-CLC2 signals (B), and a merge of the 2 signals (C). Arrows indicated the overlapping signals. Bars = 2 μm. (D), Colocalization analysis of CTL1-EGFP with mCherry-CLC2 using the ImageJ software with the colocalization finder plugin.
(TIF)

S4 Fig The expression pattern of choline transporter-like 1 enhanced green fluorescent protein (CTL1-EGFP) during apical hook development in transgenic plants expressing *CTL1pro*:*CTL1*:*EGFP* in a *ctl1* mutant background. (A–E) The fluorescence signals in the apical hook region of etiolated seedlings at 12 (A), 24 (B), 48 (C), 72 (D), 96 (E), and 120 (F) hours after germination. Arrows indicate the signals of CTL1-EGFP in the concave side of the apical hook. Bars = 100 μm. (G) The fluorescence intensity of pseudocolor images (blue-yellow-red palette) of CTL1pro:CTL1:EGFP at the apical hook of an etiolated seedling at 1 day after germination (DAG). The arrow indicates a maximum signal of CTL1 at the concave side of the apical hook. Bar = 50 μm. (H) The cortex view of fluorescence signals of CTL1pro:CTL1:EGFP in the hook region of an etiolated seedling at 1 DAG. The arrow indicates a strong signal in the concave side. Bar = 20 μm. (I) The β-glucuronidase (GUS) staining result of CTL1pro:CTL1:GUS in the apical hook region of etiolated seedling at 1 DAG. Bar = 100 μm. (J) The relative intensity of the fluorescence signals at the concave and the convex side of the apical hook. The fluorescence intensity of the concave side of 1 DAG seedling was set as 100%. The other relative values were the ratio of the fluorescence intensity against the concave signal at 1 DAG. Data are mean ± SD (*n* = 15). The raw data for panel J can be found in S1 Data.
(TIF)

S5 Fig The *ctl1* and wild-type seedlings were similarly sensitive to salicylic acid (SA). (A–E) Growth phenotype of 10-day-old wild-type (WT) and *ctl1* mutant plants grown on half-strength Murashige and Skoog (MS) medium containing 0, 10, 30, or 50 μM SA. Bars = 1 cm. (F) Relative root length (%) indicates shortening of the primary roots as a result of SA in the medium. Data are mean ± SD. Three independent experiments were performed. *n* = 10 for each experiment. The raw data for panel F can be found in S1 Data.
(TIF)

S6 Fig Relative DIRECT REPEAT5 (DR5) signals in wild type (WT) and *ctl1* mutant. (A) Relative abundance of DR5–β-glucuronidase (DR5-GUS) signals in the cotyledon tip, hypocotyl, and root parts of the WT and the *ctl1* mutants. Data are mean ± SD calculated from 5 seedlings (Student *t* test, *P < 0.05). (B) Relative intensity of DR5-green fluorescent protein (DR5-GFP) signals in the root tips of the WT and *ctl1* mutants when treated with 0, 0.1, 0.3, 0.5, or 1 μM 1-naphthylacetic acid (NAA). Data are mean ± SD calculated from 5 seedlings. (C) Relative intensity of DR5-GFP signals in the concave sides of the apical hook of the WT and the *ctl1* mutant at 1 and 3 days after germination. Data are mean ± SD calculated from 5 seedlings (Student *t* test, *P < 0.05). The raw data for panels A–C can be found in S1 Data.
(TIF)

S7 Fig Etiolated *ctl1* seedlings show reduced levels of *Arabidopsis* PM-located auxin efflux transporter PIN-formed 1 (PIN1) and *Arabidopsis* PM-located auxin efflux transporter PIN-formed 3 (PIN3) in the apical hook. Fluorescence signals in the apical section of the wild type (WT) and the ctl1 mutant indicate levels of various auxin transporters, including PIN1pro:PIN1:GFP (A and B), PIN3pro:PIN3:GFP (C and D), and AUX1pro:AUX1:GFP (E and F). Green fluorescent protein (GFP) signals in the etiolated seedlings (A–F) were examined on the first day after germination. Scale bars are 50 μm. (G) Relative fluorescence intensity of auxin transporters (PIN1, PIN3, and AUXIN RESISTANT 1 [AUX1]) in the *ctl1* mutant as compared to that in the WT (set as 100%). Data are mean ± SD calculated from 40 cells of 5 etiolated seedlings (Student *t* test, *P < 0.05). The raw data for panel G can be found in S1 Data.
(TIF)

S8 Fig The expression of brassinosteroid receptor green fluorescent protein (BRI1-GFP) did not show any difference in the wild type and the *ctl1* mutant. The GFP signals indicate the abundance of BRI1-GFP in 4-day-old primary root of the wild type (WT) (A) and the *ctl1* mutant (B). (C) Relative abundance of BRI1-GFP in the *ctl1* mutant is shown as % of the WT. Data are mean ± SD from 63 cells in 5 roots of the transgenic lines expressing BRI1-GFP in the WT versus mutant background. Three independent experiments were performed. The raw data for panel C can be found in S1 Data.
(TIF)

S9 Fig The mRNA levels of auxin transporters in the wild type and the *ctl1* mutant. Total RNA was extracted from 5-day-old wild type (WT) and *ctl1* mutant seedlings. The relative level of the WT was set as 1.0, and mutant levels were ratios against the WT level. Data are mean ± SD (*n* = 3). The raw data for this figure can be found in S1 Data.
(TIF)

S10 Fig The recycling of *Arabidopsis* PM-located auxin efflux transporter PIN-formed 2 (PIN2) is not affected in the wild type and the *ctl1* mutant. (A–D) PIN2-green fluorescent protein (GFP) localization in wild-type (WT) (A and C) and *ctl1* roots (B and D) treated with brefeldin A (BFA) for 60 minutes before (A and B) or after (C and D) washout for 90 minutes. (E) Percentage of cells with PIN2-GFP labeled BFA bodies before and after BFA washout in the WT and the *ctl1* mutant. Data are mean ± SD. Three independent experiments were performed. Five roots were used for each experiment (Student *t* test, *P < 0.05). The raw data for panel E can be found in S1 Data.
(TIF)

S11 Fig Lipid accumulation is reduced in the *ctl1* mutant. (A) Nile red staining of nonpolar lipid and polar lipid in the apical hook region of the wild type (WT) and the *ctl1* mutant. (Scale bars = 50 μm.) (B) Quantification of signal intensity of nonpolar and polar lipids as in panel A. Data are mean ± SD from 40 cells in 5 etiolated seedlings. The raw data for panel B can be found in S1 Data.
(TIF)

S12 Fig The percentages of different sphingolipids in the *ctl1* mutant when compared to the wild type. (A) Ceramide (cer); (B) Galβ1-3GalNAcβ1-3Galα1-3Galβ1-4Glcβ-Cer (Galβ1-Cer); (C) (3'-sulfo) Galβ-Cer; (D) Fucα1-2GalNAcβ1-4(NeuAcα2-8NeuAcα2–3) Galβ1-4Glcβ-Cer (Fucα1-Cer); (E) GalNAcβ1-3Galα1-3Galα1-3Galα1-3Galα1-4Galβ1-4Glcβ-Cer (GalNAcβ1-Cer); (F) Gal-Cer; (G) Manα1-3Manβ1-4Glcβ-Cer (Manα1-Cer); (H) NeuAcα2-8NeuAcα2-3Galβ1-4Glcβ-Cer (NeuAcα2-Cer); (I) GlcNAcβ1-4Manβ1-4Glcβ-Cer (d18:1/16:0), KDNα2-6Galβ1-4GlcNAcβ1-3Galβ1-4Glcβ-Cer (d18:1/26:0) and LacCer (d18:0/24:1); (J) Glc-Cer; (K) phosphatidylethanolamine (PE)-Cer; (L) phosphatidylinositol (PI)-Cer; and (M) sphingomyelins (SM). Data are mean ± SD. Four independent experiments were performed. Red asterisks indicate a significant difference (*P < 0.05, Student *t* test). The raw data can be found in S1 Data.
(TIF)

S13 Fig Sphingolipid synthesis inhibitor fumonisin B1 (FB1) blocked the formation of the apical hook and induced the opening of the apical hook. (A) Representative images of apical hooks of the wild type (WT) at 36, 60, and 84 hours after germination in the dark. Seedlings were grown on half-strength Murashige and Skoog (MS) medium containing 1 μM FB1. As FB1 was dissolved in the dimethyl sulfoxide (DMSO), the same volume of DMSO was added to the control. (B) Angles of hook curvature calculated from multiple experiments as shown in panel A. Data are mean ± SD, and 3 independent experiments were performed, with similar findings (Student *t* test, *P < 0.05). The raw data for panel B can be found in S1 Data.
(TIF)

S14 Fig Loss of the choline transporter-like 1 (CTL1) protein did not alter the morphology of organelles marked by vacuolar proton ATPase subunit VHA-a isoform 1 monomer red fluorescent protein (VHA-a1-mRFP), Syntaxin of plants 32 (SYP32)-mCherry, or *Arabidopsis* Rab homolog F2A (Rha1)-mCherry. (A and B) VHA-a1-mRFP labeled trans-Golgi network (TGN) in wild type (WT) (A) and *ctl1* (B). (C and D) SYP32-mCherry labeled Golgi apparatus in WT (C) and *ctl1* (D). (E and F) Rha1-mCherry labeled prevacuolar compartment (PVC) in WT (E) and *ctl1* (F). Bars = 2 μm. (G and H) The statistical analysis of organelle size (G) and intensity (H) in WT and *ctl1*. The organelle size and intensity were measured using the ImageJ software. Data are mean ± SD, and the significant difference was analyzed by Student *t* test. Six images from 3 roots were used. The raw data for panels G and H can be found in S1 Data.
(TIF)

S1 Table The size of AUXIN RESISTANT 1 enhanced green fluorescent protein (AUX1-EGFP)-expressing epidermal cells in cotyledons of the wild type (WT), *ctl1*, and *ctl1C*. (DOCX)

S2 Table Primers used in this study. (DOCX)

## Abbreviations

ABCB: subfamily B of ATP-binding cassete transporters
AP-3: adaptor protein complex 3
ARF-GEF: ADP-ribosylation factor-type GTPase
AUX1: AUXIN RESISTANT 1
BFA: brefeldin A
BRI1: brassinosteroid receptor
CCV: clathrin-coated vesicle of the TGN
CLC2: clathrin light chain 2
Col-0: Columbia-0
CTL1: choline transporter-like 1
DR5: DIRECT REPEAT5
EGFP: enhanced green fluorescent protein
FB1: sphingolipid synthesis inhibitor fumonisin B1
GUS: β-glucuronidase
LAX: LIKE AUX1
LAX3: Like AUX1 protein 3
mRFP: monomer red fluorescent protein
MS: Murashige and Skoog
NAA: 1-naphthylacetic acid
ORF: open reading frame
PIN1: *Arabidopsis* PM-located auxin efflux transporter PIN-formed 1
PIN2: *Arabidopsis* PM-located auxin efflux transporter PIN-formed 2
PIN3: *Arabidopsis* PM-located auxin efflux transporter PIN-formed 3
PM: plasma membrane
PVC: prevacuolar compartment
qRT-PCR: real-time quantitative reverse transcription PCR
Rha1: *Arabidopsis* Rab homolog F2A
SA: salicylic acid
SV: secretory vesicle of the TGN
SYP32: Syntaxin of plants 32
T-DNA: transfer deoxyribonucleic acid
TGN/EE: trans-Golgi network/early endosome
UPLC-MS: ultra-performance liquid chromatography coupled with mass spectrometry
VHA-a1: vacuolar proton ATPase subunit VHA-a isoform 1
Wm: wortmannin
WT: wild type
