# Supplementary figures and images for: Arabidopsis choline transporter-like 1 (CTL1) regulates secretory trafficking of auxin transporters to control seedling growth
Source: PLoS Biol. 2017 Dec 28;15(12):e2004310. doi: 10.1371/journal.pbio.2004310 (PMC5746207; doi:10.1371/journal.pbio.2004310)

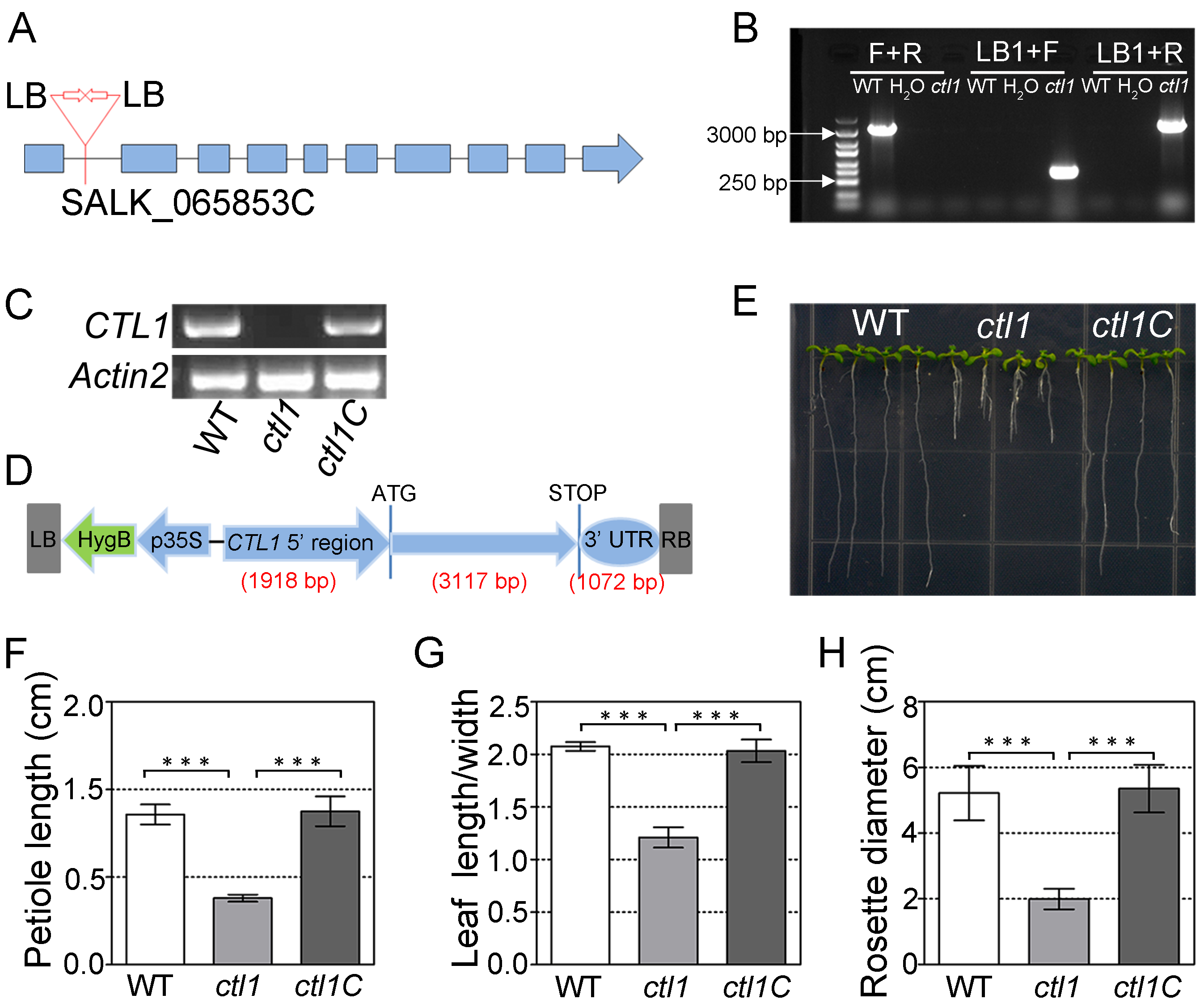

Supplement: S1 Fig — (A) Schematic representation of the CTL1 gene. The T-DNA insertion site is located in the first intron (281-bp downstream of the ATG start codon) and is indicated by a triangle. Solid boxes and lines indicate the exons and introns, respectively. (B) Confirmation of the T-DNA insertion in the ctl1 mutant by PCR using the forward primer (F) and reverse primer (R) of CTL1 paired to the T-DNA border primer LB1. (C) Reverse transcription PCR (RT-PCR) analysis of CTL1 mRNA from 10-day-old wild type (WT), ctl1, and ctl1 complemented with a genomic fragment of the CTL1 gene (ctl1C). ACTIN2 mRNA was analyzed as a loading control. (D) Schematic model of the T-DNA region of vector pCAMBIA-1300 containing a genomic fragment of the CTL1 gene. (E) The growth phenotype of 10-day-old WT, ctl1, and ctl1C. (F–H) Quantitative analysis of the petiole length of rosette leaves (F), the ratio of leaf length to width (G), and the rosette diameter (H) in 30-day-old WT, ctl1 and ctl1C grown in the soil. Data are mean ± SD, and significant differences are indicated as ***P < 0.01 (n = 5, Student t test). The raw data for panels F–H can be found in S1 Data. (TIF) [file pbio.2004310.s003.tif]

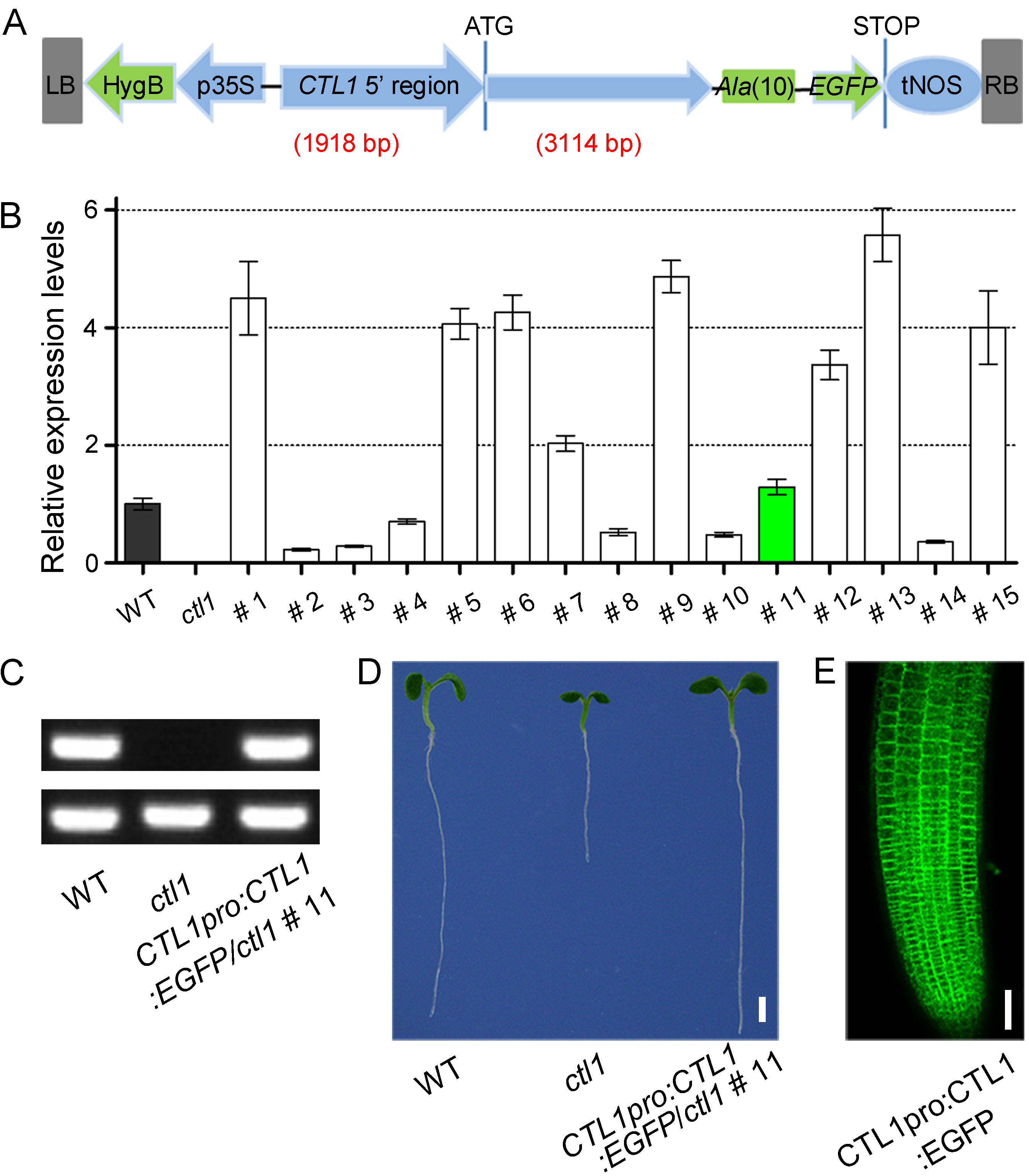

Supplement: S2 Fig — (A) Schematic model of the transfer deoxyribonucleic acid (T-DNA) region of vector pCAMBIA-1300 containing a genomic fragment of the choline transporter-like 1 (CTL1) gene fused with enhanced green fluorescent protein (EGFP). A 10-alanine (Ala) linker sequence was inserted between CTL1 and EGFP. (B) Real-time quantitative reverse transcription PCR (qRT-PCR) analysis of CTL1 mRNA levels in 10-day-old wild type (WT), ctl1, and lines expressing CTL1pro:CTL1:EGFP in the ctl1 mutant background. Relative expression levels were calculated as the ratio of CTL1 in various seedlings to that in WT. Data are mean ± SD (n = 3). Fifteen CTL1pro:CTL1:EGFP/ctl1 lines were selected for qRT-PCR analysis, and one of them, #11, with CTL1 levels similar to those of the WT, was used for further studies. (C) RT-PCR analysis of CTL1 mRNA in 10-day-old ctl1 mutant and CTL1pro:CTL1:EGFP-transformed line (CTL1pro:CTL1:EGFP/ctl1 #11). ACTIN2 mRNA was analyzed as a loading control. (D) Growth phenotype of 5-day-old WT, ctl1, and CTL1pro:CTL1:EGFP/ctl1 #11 grown on half-strength Murashige and Skoog (MS) solid medium. Bar = 2 mm. (E) Fluorescence signals in the primary root of a 4-day-old CTL1pro:CTL1:EGFP/ctl1 #11 seedling grown on half-strength MS solid medium. Bar = 50 μm. The raw data for panel B can be found in S1 Data. (TIF) [file pbio.2004310.s004.tif]

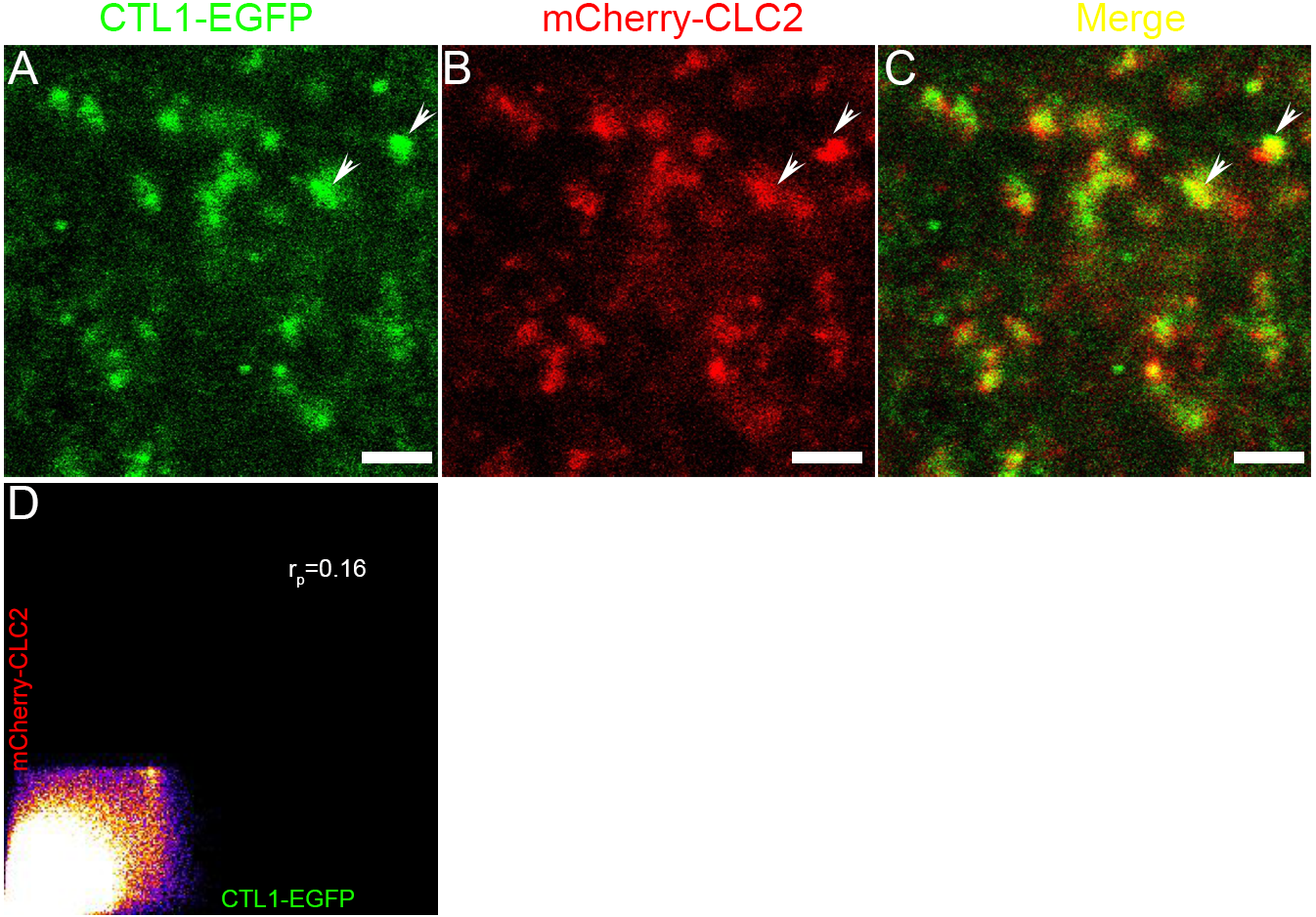

Supplement: S3 Fig — A representative confocal image of CTL1-EGFP signals (A), mCherry-CLC2 signals (B), and a merge of the 2 signals (C). Arrows indicated the overlapping signals. Bars = 2 μm. (D), Colocalization analysis of CTL1-EGFP with mCherry-CLC2 using the ImageJ software with the colocalization finder plugin. (TIF) [file pbio.2004310.s005.tif]

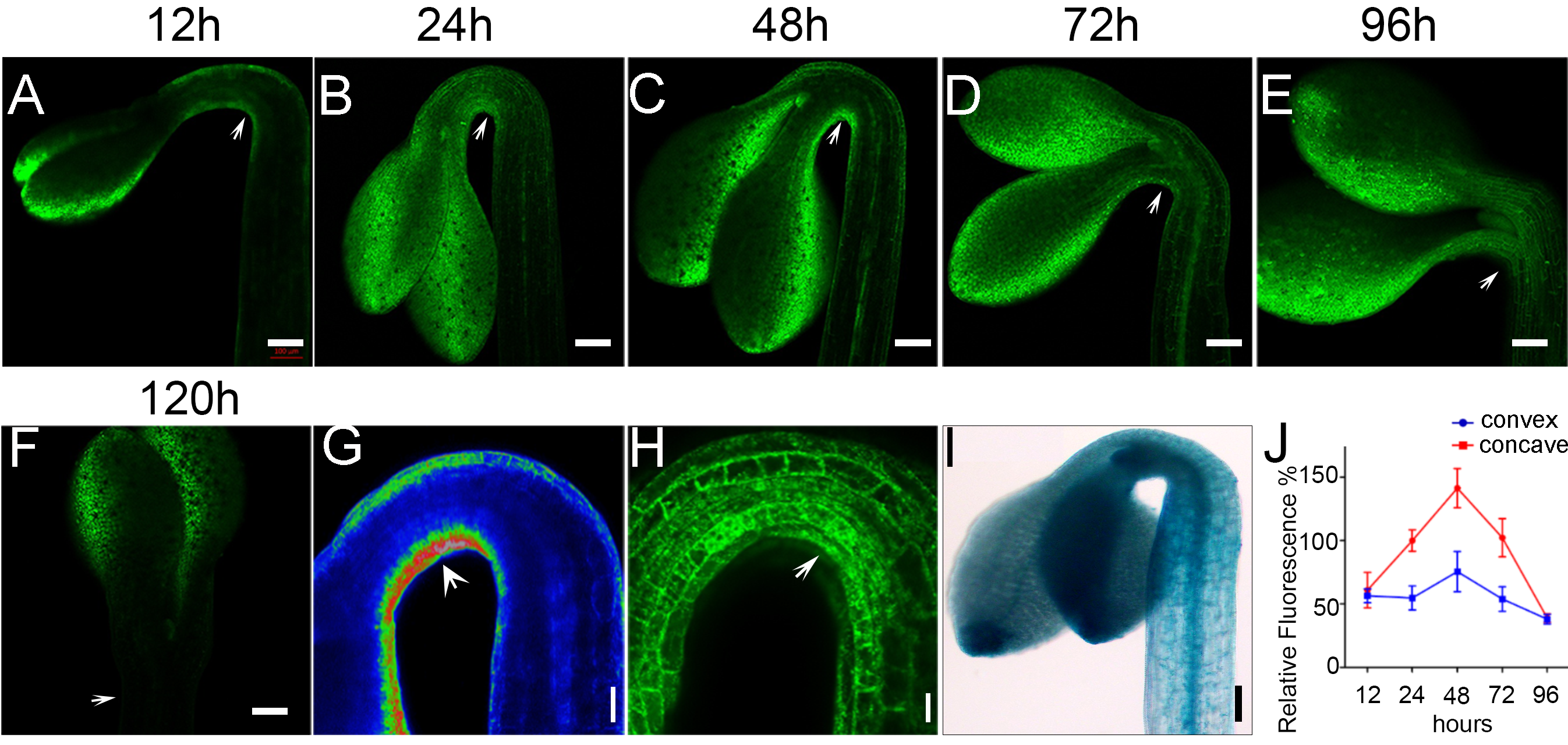

Supplement: S4 Fig — (A–E) The fluorescence signals in the apical hook region of etiolated seedlings at 12 (A), 24 (B), 48 (C), 72 (D), 96 (E), and 120 (F) hours after germination. Arrows indicate the signals of CTL1-EGFP in the concave side of the apical hook. Bars = 100 μm. (G) The fluorescence intensity of pseudocolor images (blue-yellow-red palette) of CTL1pro:CTL1:EGFP at the apical hook of an etiolated seedling at 1 day after germination (DAG). The arrow indicates a maximum signal of CTL1 at the concave side of the apical hook. Bar = 50 μm. (H) The cortex view of fluorescence signals of CTL1pro:CTL1:EGFP in the hook region of an etiolated seedling at 1 DAG. The arrow indicates a strong signal in the concave side. Bar = 20 μm. (I) The β-glucuronidase (GUS) staining result of CTL1pro:CTL1:GUS in the apical hook region of etiolated seedling at 1 DAG. Bar = 100 μm. (J) The relative intensity of the fluorescence signals at the concave and the convex side of the apical hook. The fluorescence intensity of the concave side of 1 DAG seedling was set as 100%. The other relative values were the ratio of the fluorescence intensity against the concave signal at 1 DAG. Data are mean ± SD (n = 15). The raw data for panel J can be found in S1 Data. (TIF) [file pbio.2004310.s006.tif]

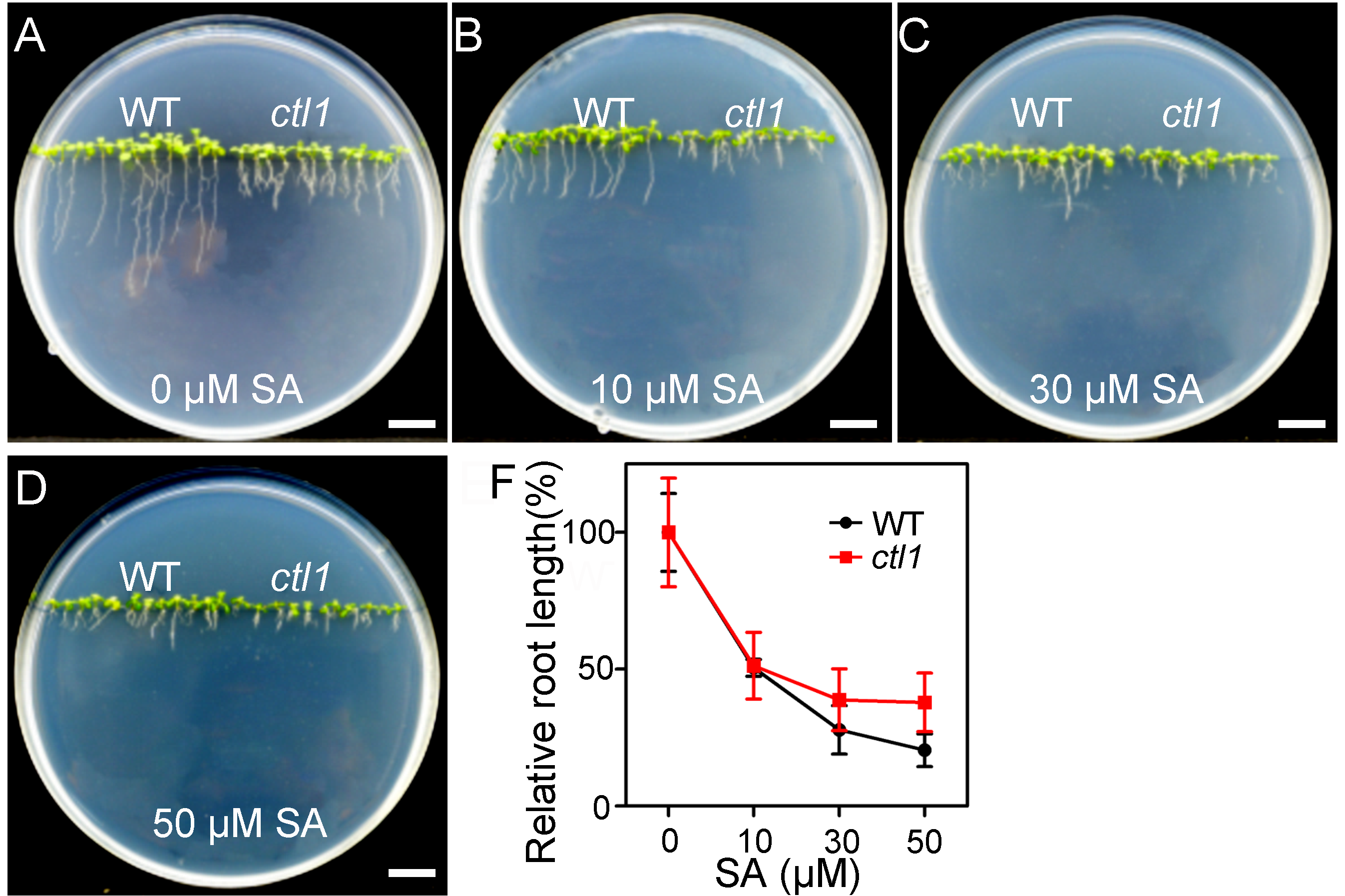

Supplement: S5 Fig — (A–E) Growth phenotype of 10-day-old wild-type (WT) and ctl1 mutant plants grown on half-strength Murashige and Skoog (MS) medium containing 0, 10, 30, or 50 μM SA. Bars = 1 cm. (F) Relative root length (%) indicates shortening of the primary roots as a result of SA in the medium. Data are mean ± SD. Three independent experiments were performed. n = 10 for each experiment. The raw data for panel F can be found in S1 Data. (TIF) [file pbio.2004310.s007.tif]

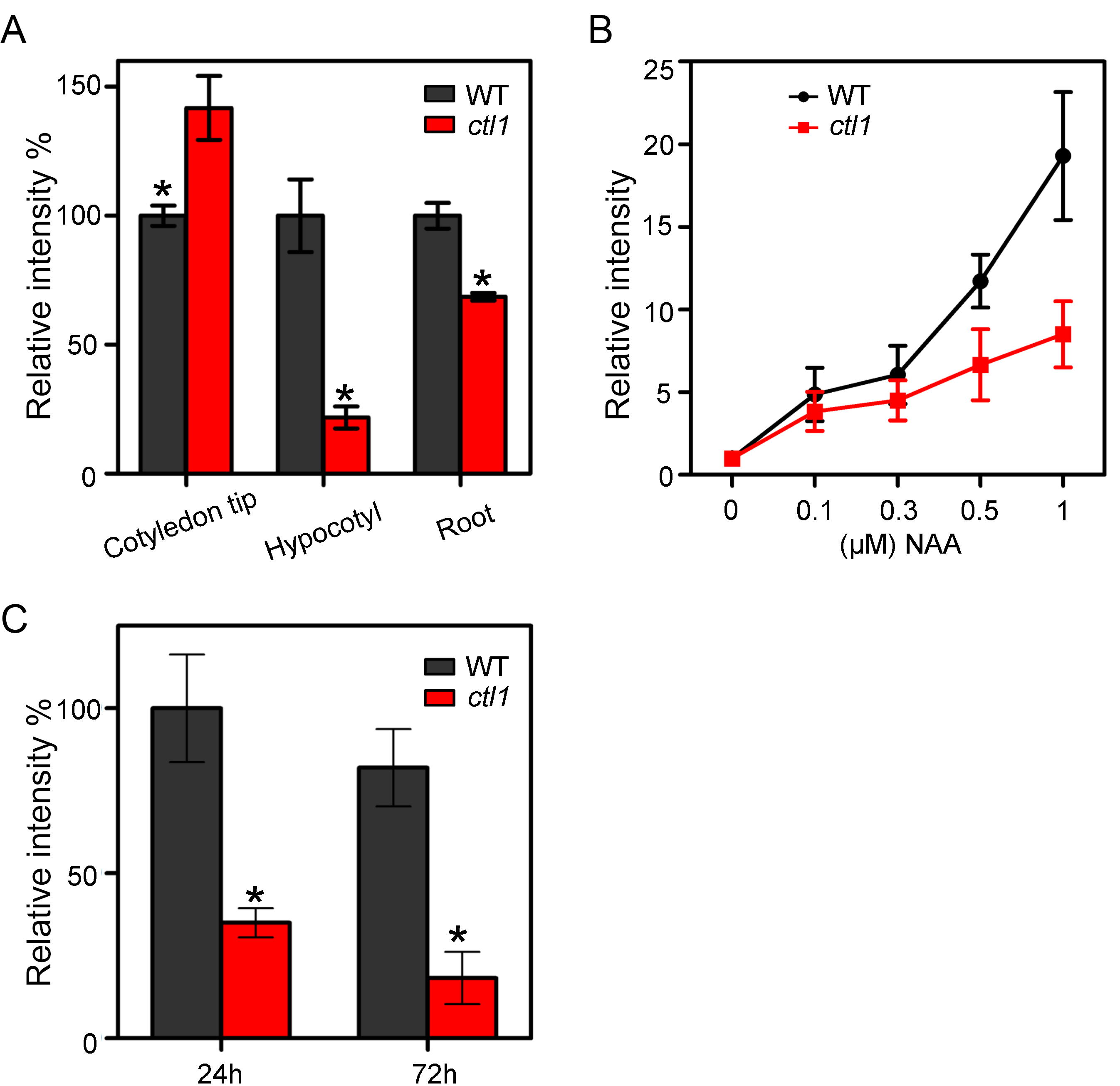

Supplement: S6 Fig — (A) Relative abundance of DR5–β-glucuronidase (DR5-GUS) signals in the cotyledon tip, hypocotyl, and root parts of the WT and the ctl1 mutants. Data are mean ± SD calculated from 5 seedlings (Student t test, *P < 0.05). (B) Relative intensity of DR5-green fluorescent protein (DR5-GFP) signals in the root tips of the WT and ctl1 mutants when treated with 0, 0.1, 0.3, 0.5, or 1 μM 1-naphthylacetic acid (NAA). Data are mean ± SD calculated from 5 seedlings. (C) Relative intensity of DR5-GFP signals in the concave sides of the apical hook of the WT and the ctl1 mutant at 1 and 3 days after germination. Data are mean ± SD calculated from 5 seedlings (Student t test, *P < 0.05). The raw data for panels A–C can be found in S1 Data. (TIF) [file pbio.2004310.s008.tif]

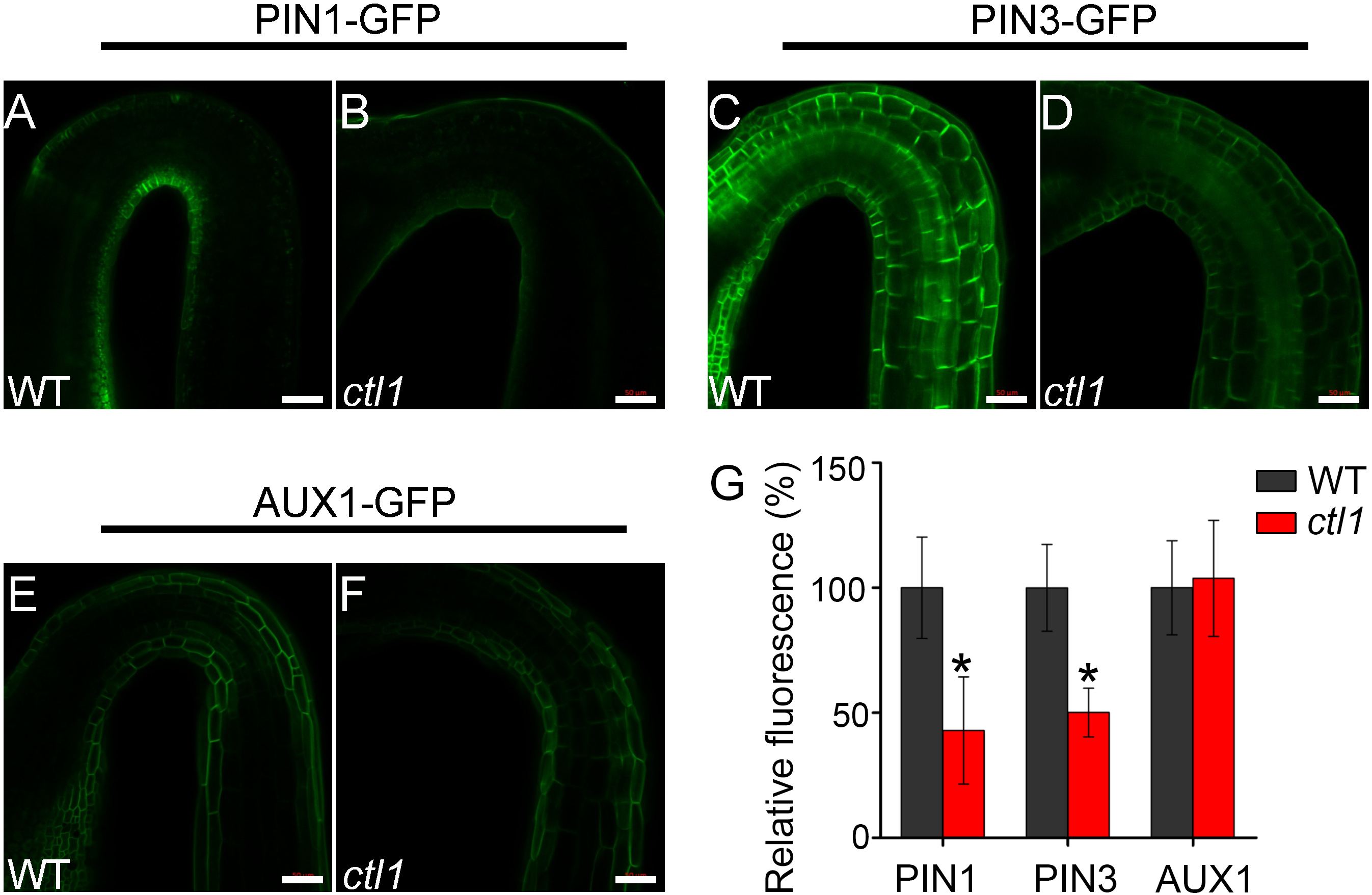

Supplement: S7 Fig — Fluorescence signals in the apical section of the wild type (WT) and the ctl1 mutant indicate levels of various auxin transporters, including PIN1pro:PIN1:GFP (A and B), PIN3pro:PIN3:GFP (C and D), and AUX1pro:AUX1:GFP (E and F). Green fluorescent protein (GFP) signals in the etiolated seedlings (A–F) were examined on the first day after germination. Scale bars are 50 μm. (G) Relative fluorescence intensity of auxin transporters (PIN1, PIN3, and AUXIN RESISTANT 1 [AUX1]) in the ctl1 mutant as compared to that in the WT (set as 100%). Data are mean ± SD calculated from 40 cells of 5 etiolated seedlings (Student t test, *P < 0.05). The raw data for panel G can be found in S1 Data. (TIF) [file pbio.2004310.s009.tif]

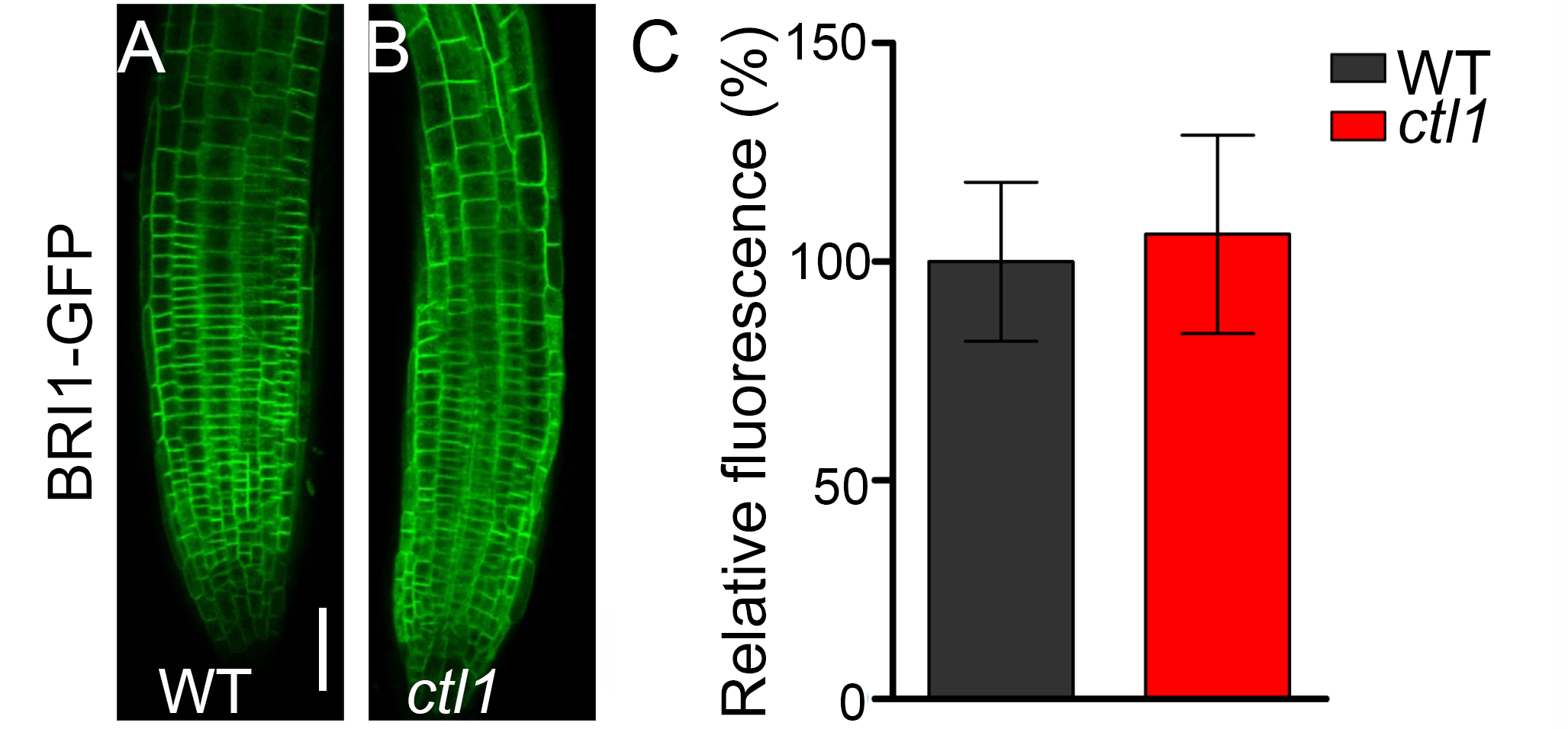

Supplement: S8 Fig — The GFP signals indicate the abundance of BRI1-GFP in 4-day-old primary root of the wild type (WT) (A) and the ctl1 mutant (B). (C) Relative abundance of BRI1-GFP in the ctl1 mutant is shown as % of the WT. Data are mean ± SD from 63 cells in 5 roots of the transgenic lines expressing BRI1-GFP in the WT versus mutant background. Three independent experiments were performed. The raw data for panel C can be found in S1 Data. (TIF) [file pbio.2004310.s010.tif]

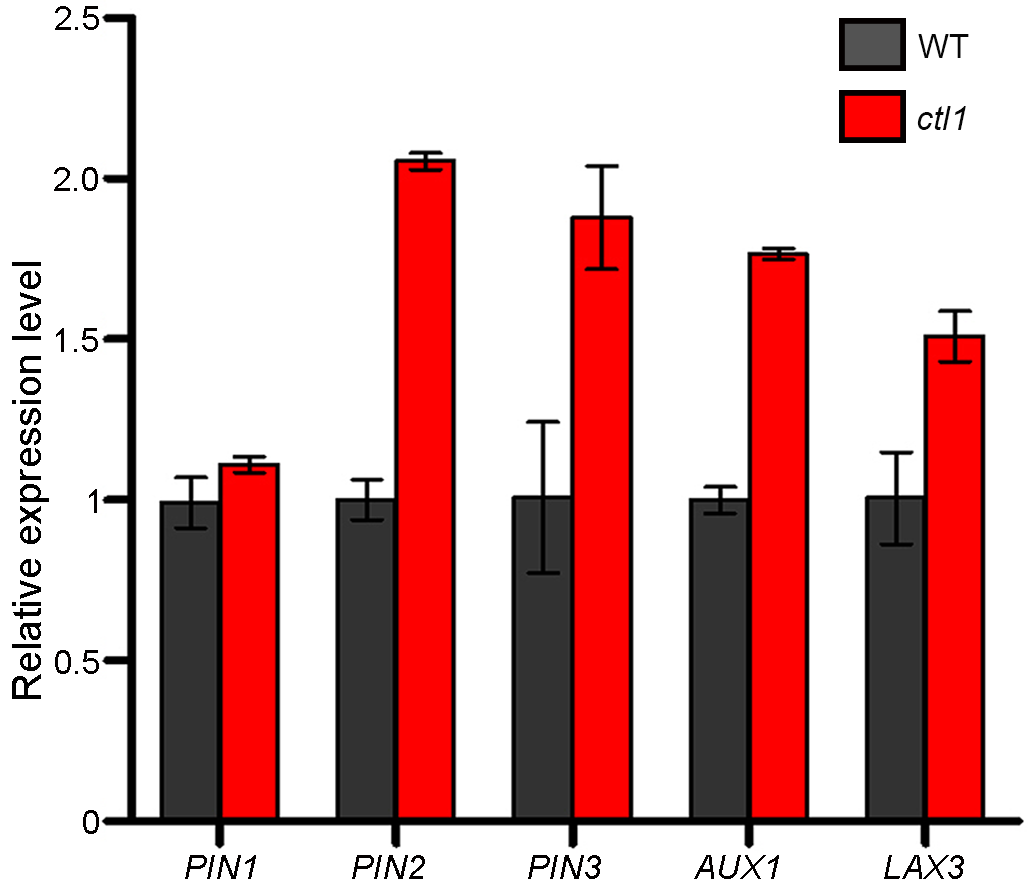

Supplement: S9 Fig — Total RNA was extracted from 5-day-old wild type (WT) and ctl1 mutant seedlings. The relative level of the WT was set as 1.0, and mutant levels were ratios against the WT level. Data are mean ± SD (n = 3). The raw data for this figure can be found in S1 Data. (TIF) [file pbio.2004310.s011.tif]

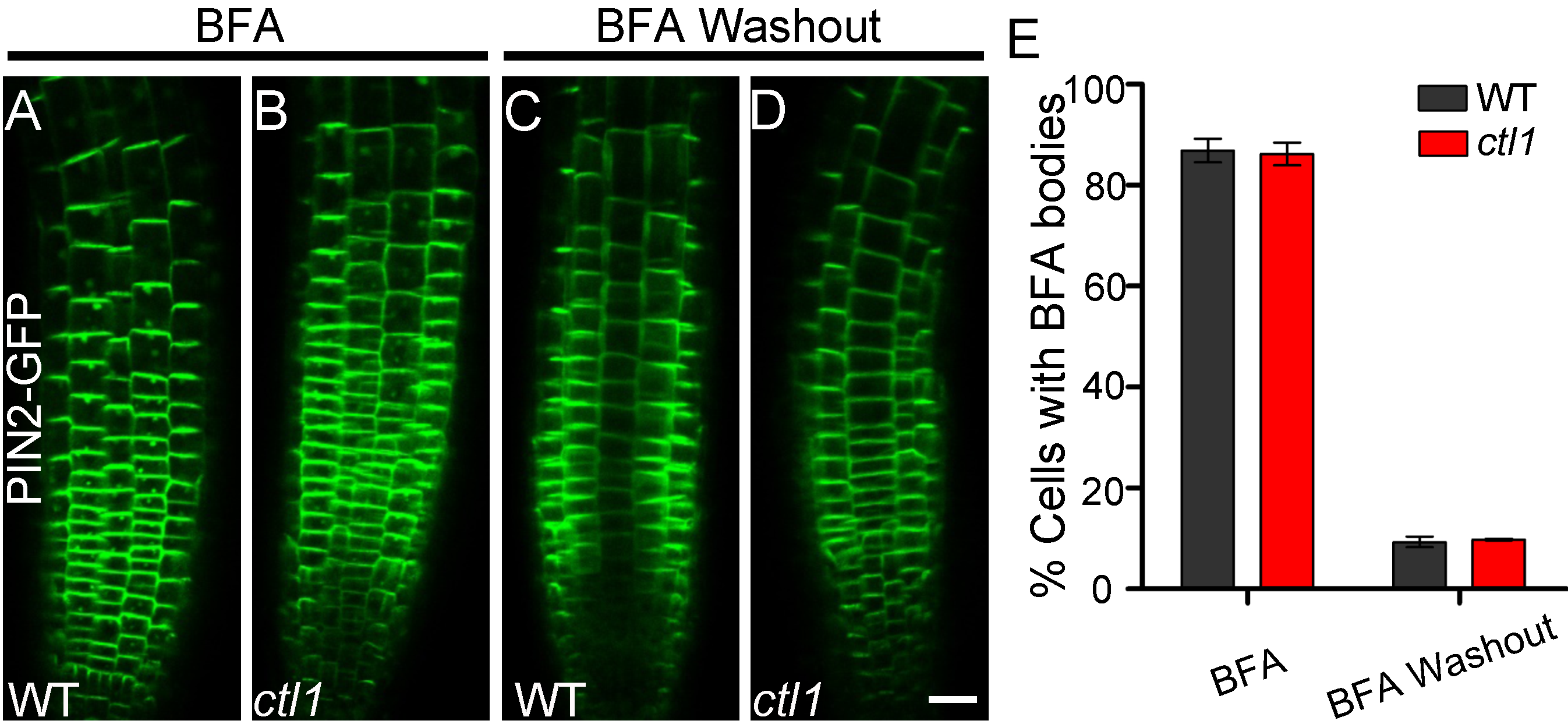

Supplement: S10 Fig — (A–D) PIN2-green fluorescent protein (GFP) localization in wild-type (WT) (A and C) and ctl1 roots (B and D) treated with brefeldin A (BFA) for 60 minutes before (A and B) or after (C and D) washout for 90 minutes. (E) Percentage of cells with PIN2-GFP labeled BFA bodies before and after BFA washout in the WT and the ctl1 mutant. Data are mean ± SD. Three independent experiments were performed. Five roots were used for each experiment (Student t test, *P < 0.05). The raw data for panel E can be found in S1 Data. (TIF) [file pbio.2004310.s012.tif]

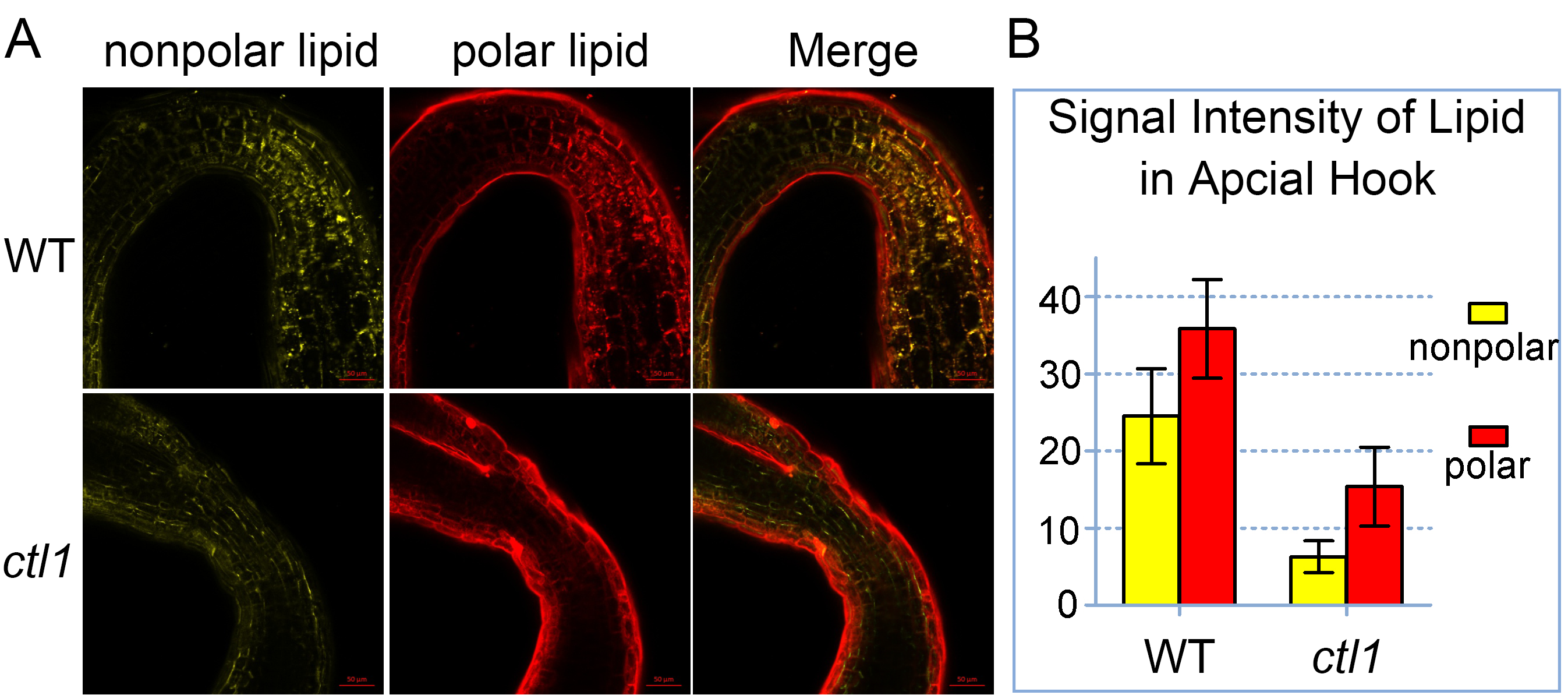

Supplement: S11 Fig — (A) Nile red staining of nonpolar lipid and polar lipid in the apical hook region of the wild type (WT) and the ctl1 mutant. (Scale bars = 50 μm.) (B) Quantification of signal intensity of nonpolar and polar lipids as in panel A. Data are mean ± SD from 40 cells in 5 etiolated seedlings. The raw data for panel B can be found in S1 Data. (TIF) [file pbio.2004310.s013.tif]

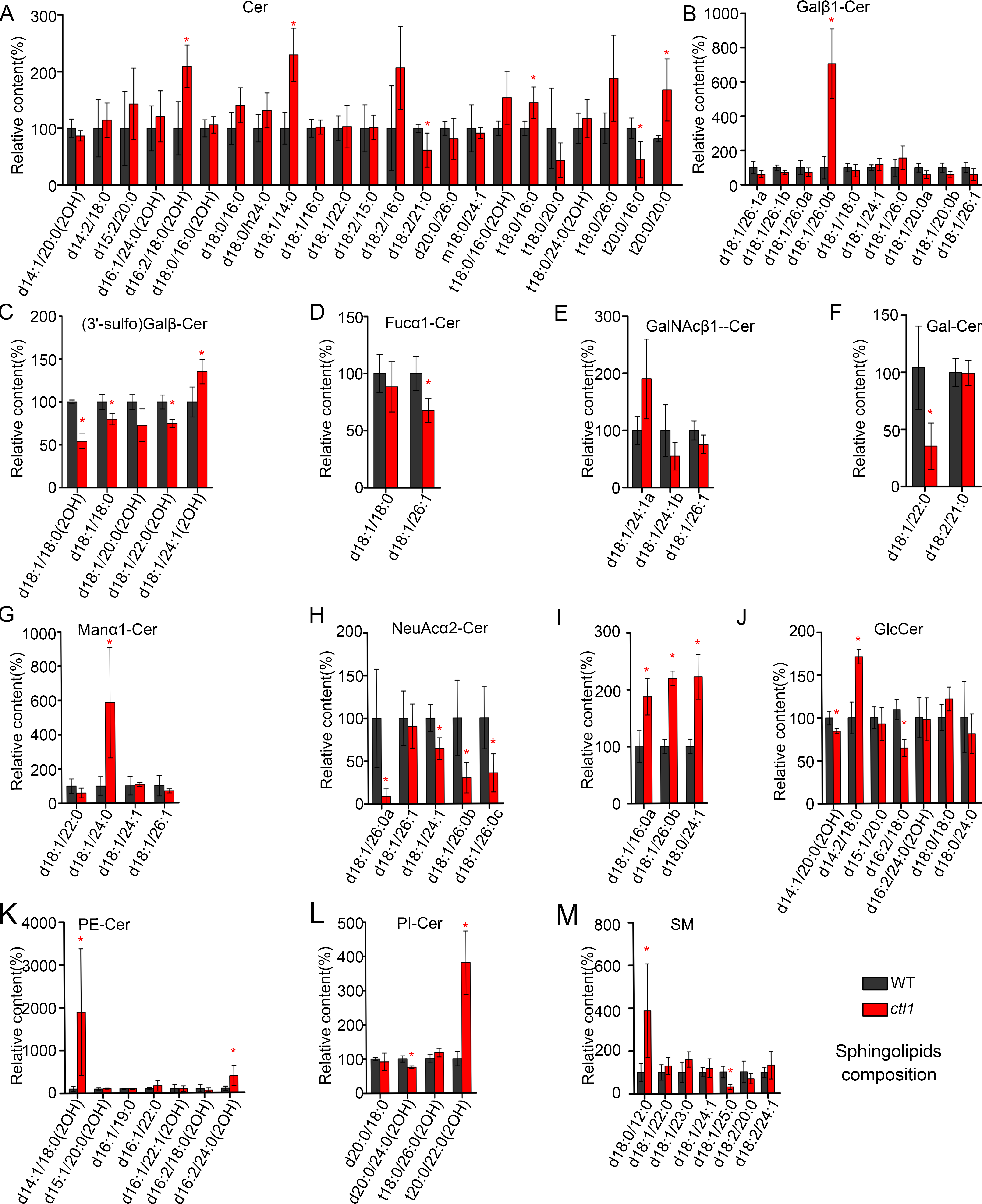

Supplement: S12 Fig — (A) Ceramide (cer); (B) Galβ1-3GalNAcβ1-3Galα1-3Galβ1-4Glcβ-Cer (Galβ1-Cer); (C) (3'-sulfo) Galβ-Cer; (D) Fucα1-2GalNAcβ1-4(NeuAcα2-8NeuAcα2–3) Galβ1-4Glcβ-Cer (Fucα1-Cer); (E) GalNAcβ1-3Galα1-3Galα1-3Galα1-3Galα1-4Galβ1-4Glcβ-Cer (GalNAcβ1-Cer); (F) Gal-Cer; (G) Manα1-3Manβ1-4Glcβ-Cer (Manα1-Cer); (H) NeuAcα2-8NeuAcα2-3Galβ1-4Glcβ-Cer (NeuAcα2-Cer); (I) GlcNAcβ1-4Manβ1-4Glcβ-Cer (d18:1/16:0), KDNα2-6Galβ1-4GlcNAcβ1-3Galβ1-4Glcβ-Cer (d18:1/26:0) and LacCer (d18:0/24:1); (J) Glc-Cer; (K) phosphatidylethanolamine (PE)-Cer; (L) phosphatidylinositol (PI)-Cer; and (M) sphingomyelins (SM). Data are mean ± SD. Four independent experiments were performed. Red asterisks indicate a significant difference (*P < 0.05, Student t test). The raw data can be found in S1 Data. (TIF) [file pbio.2004310.s014.tif]

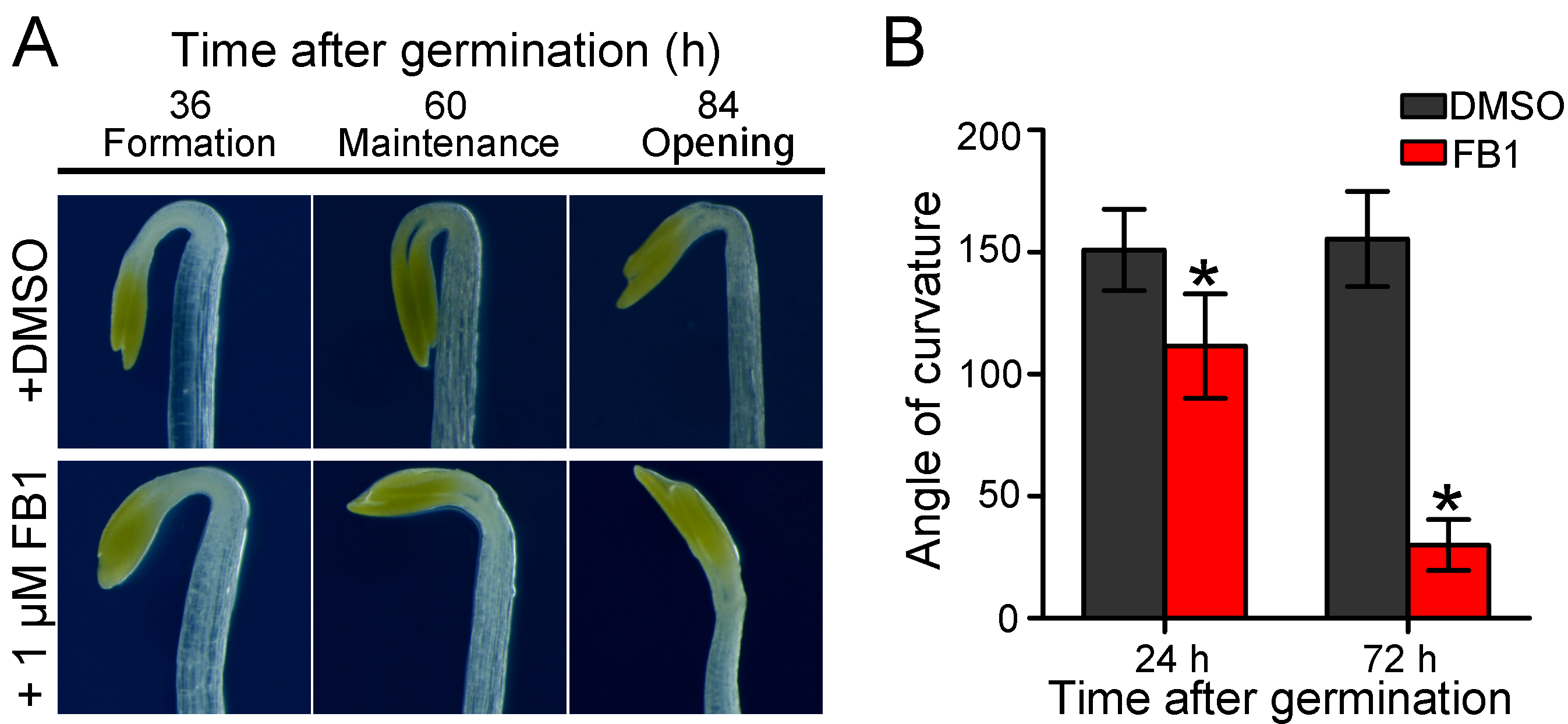

Supplement: S13 Fig — (A) Representative images of apical hooks of the wild type (WT) at 36, 60, and 84 hours after germination in the dark. Seedlings were grown on half-strength Murashige and Skoog (MS) medium containing 1 μM FB1. As FB1 was dissolved in the dimethyl sulfoxide (DMSO), the same volume of DMSO was added to the control. (B) Angles of hook curvature calculated from multiple experiments as shown in panel A. Data are mean ± SD, and 3 independent experiments were performed, with similar findings (Student t test, *P < 0.05). The raw data for panel B can be found in S1 Data. (TIF) [file pbio.2004310.s015.tif]

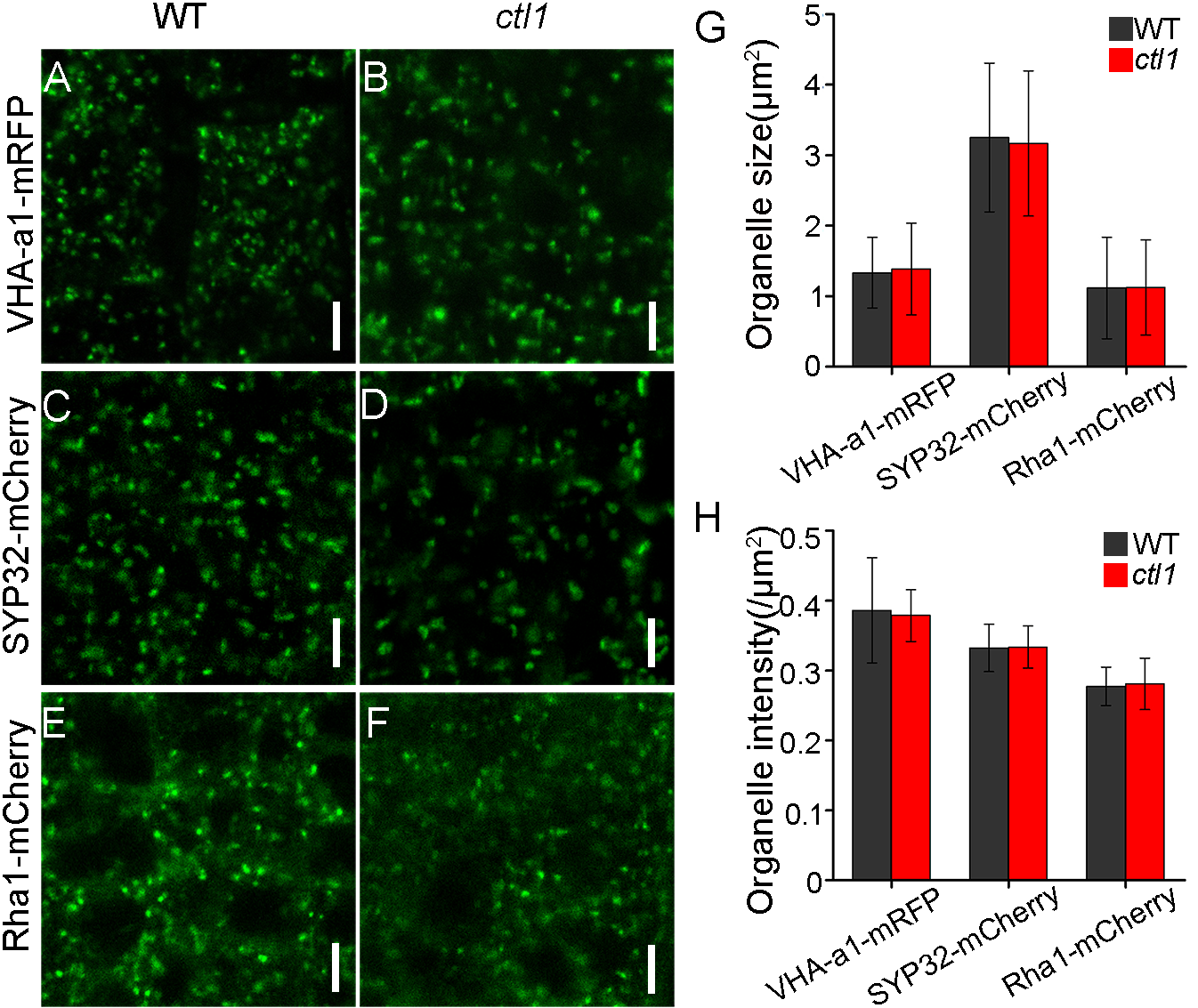

Supplement: S14 Fig — (A and B) VHA-a1-mRFP labeled trans-Golgi network (TGN) in wild type (WT) (A) and ctl1 (B). (C and D) SYP32-mCherry labeled Golgi apparatus in WT (C) and ctl1 (D). (E and F) Rha1-mCherry labeled prevacuolar compartment (PVC) in WT (E) and ctl1 (F). Bars = 2 μm. (G and H) The statistical analysis of organelle size (G) and intensity (H) in WT and ctl1. The organelle size and intensity were measured using the ImageJ software. Data are mean ± SD, and the significant difference was analyzed by Student t test. Six images from 3 roots were used. The raw data for panels G and H can be found in S1 Data. (TIF) [file pbio.2004310.s016.tif]
