# Supplementary material for: Arabidopsis choline transporter-like 1 (CTL1) regulates secretory trafficking of auxin transporters to control seedling growth
Source: PLoS Biol. 2017 Dec 28;15(12):e2004310. doi: 10.1371/journal.pbio.2004310 (PMC5746207; doi:10.1371/journal.pbio.2004310)
Supplement: S1 Table — (DOCX) [file pbio.2004310.s017.docx]

**Supplemental Table 1.** The size of AUX1-EGFP-expressing epidermal cells in cotyledons of wild type (WT), *ctl1*, and *ctl1C*.

| Seedling | Cell size (μm^2^) | n | Relative size to WT |
| --- | --- | --- | --- |
| WT | 3.6 ± 0.7 | 18 | 100% |
| *ctl1* | 1.8 ± 0.6 | 20 | 49% |
| *ctl1C* | 3.5 ± 0.7 | 15 | 99% |

The epidermis was detached from the cotyledons of 5-day-old seedlings grown on half-strength MS solid medium. Data are mean ± SD.
