# Supplementary material for: Arabidopsis choline transporter-like 1 (CTL1) regulates secretory trafficking of auxin transporters to control seedling growth
Source: PLoS Biol. 2017 Dec 28;15(12):e2004310. doi: 10.1371/journal.pbio.2004310 (PMC5746207; doi:10.1371/journal.pbio.2004310)
Supplement: S2 Table — (DOCX) [file pbio.2004310.s018.docx]

**Supplemental Table 2. Primers used in this study.**

| Names | Primer sequences | Purposes |
| --- | --- | --- |
| CTL1-KpnI-F | 5'-GGGGTACCCTACAAAACTAAGTC  GTGCTCTGGT-3' | Construction of  pCTL1:CTL1:GFP |
| CTL1-SmaI-R | 5'-TCCCCCGGGCGTGAGTAAGACTC  TGAACCTCTTC-3' |  |
| CTL1-XbaI-F | 5'-GCTCTAGACTCAATTGTAAATACA  AAGACAGATG-3' |  |
| CTL1-XbaI-R | 5'-GCTCTAGAGAGCAAGGAAAAGGA  TTTTACC-3' |  |
| EGFP-XbaI-R | 5'-GCTCTAGATTACTTGTACAGCTC-3' |  |
| SLBa1 | 5'-TGGTTCACGTAGTGGGCCATCG-3' | Identification of homozygote |
| CTL1-F | 5'-ATGAGAGGACCTTTAGGAGCAG-3' |  |
| CTL1-R | 5'-TCAGTGAGTAAGACTCTGAACCT-3' |  |
| GUS-F | 5'-GCTCTAGACTACAAAACTAAGTCG  TGCTCTGGT-3' | Construction of pCTL1:CTL1:GUS |
| GUS-R | 5'-TCCCCCGGGGGAGTGAGTAAGAC  TCTGAACCTCTTCC-3' |  |
| LAX3-KpnI-F | 5'-GGGGTACCTTTTATCTGTCTATTT-3' | Construction of pLAX3:LAX3:GFP |
| LAX3-SmaI-R | 5'-TCCCCCGGGCTGGCTTGTGAGG-3' |  |
| EGFP-XbaI-R | 5'-GCTCTAGATTACTTGTACAGCTC-3' |  |
| Actin-F | 5'-ACTCTCCCGCTATGTATGTCGCC-3' | qRT-PCR analysis of *Actin2* |
| Actin-R | 5'-ATTTCCCGCTCTGCTGTTGTGGT-3' |  |
| CTL1-F | 5'-TGGCAGCAGATGGGTGGTGT-3' | qRT-PCR analysis of *CTL1* expression |
| CTL1-R | 5'-TGGAACAAGGCCTCCACAAACG-3' |  |
| PIN1-F | 5'-TCGTCGCTCTCTTCGCCGTT-3' | qRT-PCR analysis of auxin transporters expression in WT and *ctl1* |
| PIN1-R | 5'-TGCGGCTGAGTTTGCACCAG-3' |  |
| PIN2-F | 5'-TCGTTGCGGTTTTCGCGGTT-3' |  |
| PIN2-R | 5'-CCTCTGCGGCTAAACGCCTG-3' |  |
| PIN3-F | 5'-GGTTTCCCCGGTGGTCGTCT-3' |  |
| PIN3-R | 5'-TCGGAGCCGGATAAGACCCG-3' |  |
| AUX1-F | 5'-ACCTTTGGAGGTCACGCGGTT-3' |  |
| AUX1-R | 5'-GTGCGTCTCCGAAAGCCCAG-3' |  |
| LAX3-F | 5'-TGGTCATTCCTCGGACTCGCT-3' |  |
| LAX3-R | 5'-TCTCCACTGTGACGGCGTGG-3' |  |
